# London dispersion as important factor for the stabilization of (*Z*)-azobenzenes in the presence of hydrogen bonding

**DOI:** 10.3762/bjoc.14.106

**Published:** 2018-05-29

**Authors:** Andreas H Heindl, Raffael C Wende, Hermann A Wegner

**Affiliations:** 1 Institut für Organische Chemie, Justus-Liebig-Universität Gießen, Heinrich-Buff-Ring 17, 35392 Gießen, Germany

**Keywords:** azobenzene, hydrogen bonding, London dispersion, molecular switches

## Abstract

The understanding and control of the light-induced isomerization of azobenzenes as one of the most important classes of molecular switches is crucial for the design of light-responsive materials using this entity. Herein, we present the stabilization of metastable (*Z*)-azobenzenes by London dispersion interactions, even in the presence of comparably stronger hydrogen bonds in various solvents. The *Z*→*E* isomerization rates of several *N*-substituted 4,4′-bis(4-aminobenzyl)azobenzenes were measured. An intramolecular stabilization was observed and explained by the interplay of intramolecular amide and carbamate hydrogen bonds as well as London dispersion interactions. Whereas in toluene, 1,4-dioxane and *tert*-butyl methyl ether the hydrogen bonds dominate, the variation in stabilization of the different substituted azobenzenes in dimethyl sulfoxide can be rationalized by London dispersion interactions. These findings were supported by conformational analysis and DFT computations and reveal low-energy London dispersion forces to be a significant factor, even in the presence of hydrogen bonds.

## Introduction

The photo-controlled *E*→*Z* isomerization of azobenzene has been known for decades [1] and has originated a wide field of applications in recent years. This molecular switch has been utilized inter alia in the rising field of photopharmacology [2–3], the manipulation of biomolecular processes [4–6] as well as in molecular machinery [7–8] and materials science [9–11]. Azobenzenes are highly stable, easily synthesized [12] and show reversible isomerization from the thermally stable *E-* to the *Z*-isomer upon irradiation with UV light. The metastable *Z*-azobenzene re-isomerizes to the *E-*conformer either thermally or upon irradiation with visible light [13–14]. Interestingly, the thermal stability of azobenzene isomers can be reversed by the incorporation of azobenzene units in macrocyclic arrangements [15]. For example, the groups of Tamaoki [16] and Herges [17] reported azobenzophanes that isomerize thermally to their energetically lower *Z*-conformations from their corresponding higher energy *E*-isomers. Moreover, our group presented a highly strained bisazobenzophane that was found to be stable exclusively in its (*Z,Z*)-form. Consequently, no isomerization, neither photochemically nor thermally, to its (*E,Z*)- or (*E,E*)-state occurred on the observed time scale [18].

**Scheme 1:**
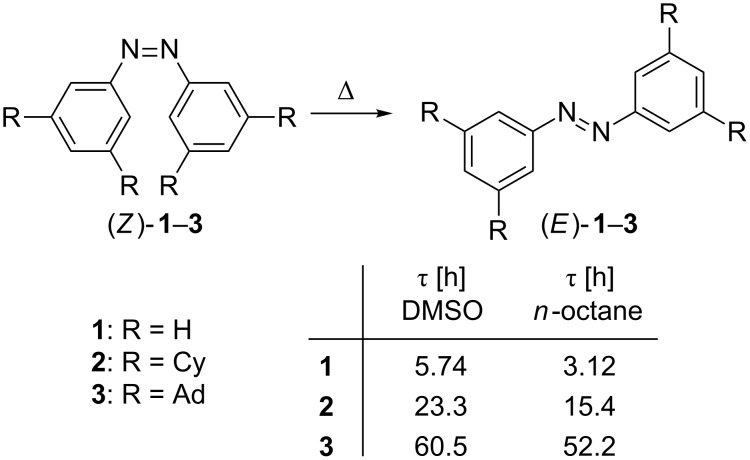
Half-lives for the thermal *Z*→*E* isomerization of all-*meta*-alkyl-substituted azobenzenes **1**–**3** in DMSO and *n*-octane at 53.2 °C (examples taken from ref. [23], Cy = cyclohexyl, Ad = adamantyl).

The thermal half-lives of acyclic (*Z*)-azobenzenes can be prolonged dramatically by *ortho*-fluorine substitution, resulting in half-lives of up to two years at room temperature [19]. These highly thermally stable (*Z*)-*ortho*-fluoroazobenzenes can be re-isomerized almost instantaneously in an electrocatalytic fashion [20]. Furthermore, (*Z*)-azobenzenes can also be stabilized without changing their electronic configuration by attractive London dispersion (LD) interactions [21–22]. In a recent study, we reported that all-*meta*-alkyl-substituted (*Z*)-azobenzenes increase in stability with increasingly larger substituents (Scheme 1) [23]. Supported by density functional theory (DFT) computations, attractive LD forces were identified as the origin of this stability trend. Based on this study, LD interactions represent a valuable tool for the design of novel azobenzene photoswitches [24]. Herein we provide further evidence for the importance of LD as unneglectable stabilizing element in controlling interactions in functionalized molecules.

**Scheme 2:**
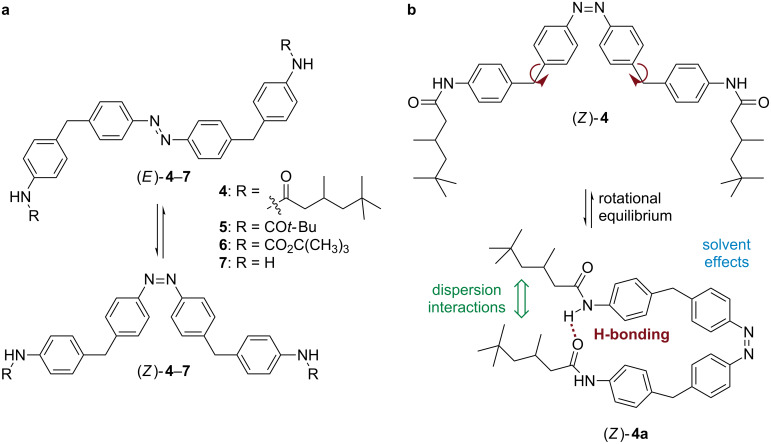
Isomerization of *N*-substituted *Z*-azobenzenes (a). Rotational equilibrium of (*Z*)-**4** allowing intramolecular interactions (b).

During studies on cyclobisazodiphenylmethane [18] we noticed that the bulky (*Z*)-4,4’-bis[4-(3,5,5-trimethylhexanoylamino)benzyl]azobenzene (**4**, Scheme 2a) showed an unexpectedly increased thermal half-life compared to other azobenzenes **5**–**7** in *tert*-butyl methyl ether (TBME) as solvent. This observation is surprising, since azobenzenes **4**–**7** are electronically very similar as the methylene linker prevents conjugation of the *N*-aryl and the azobenzene moieties. At first thought, the large spatial separation of the *N*-substituted moieties in (*Z*)-**4**–**7** make intramolecular stabilizing interactions unlikely. Nevertheless, it can be envisioned that the freely rotatable Ar–CH_2_–Ar units should allow the formation of close proximity conformers of (*Z*)-**4a** in solution, in which attractive interactions, such as hydrogen bonding and London dispersion, may indeed become possible (Scheme 2b) [25]. To further investigate this phenomenon, the rates of the thermal *Z*→*E* isomerization of azobenzenes **4**–**7** were determined in different solvents and at different temperatures (for syntheses, see ref. [18] and experimental section).

## Results and Discussion

To further investigate the prior observations, the thermal *Z*→*E* isomerization rates of azobenzenes **4**–**7** were determined by UV–vis spectroscopy in several solvents at 25 °C and 35 °C, respectively. The lowest thermal isomerization rates were found for the crowded 4,4’-bis[4-(3,5,5,-trimethylhexanoylamino)benzyl]azobenzene (**4**, Table 1, entry 1), expressed in the longest half-lives τ. However, in the highly polar solvent dimethyl sulfoxide (DMSO) all azoamides (**4**, **5**, Table 1, entries 1 and 2) as well as the azocarbamate **6** (Table 1, entry 3) showed very similar isomerization rates. Additionally, nearly the same half-lives were observed in all solvents for the *Z*→*E* isomerizations of azoamine **7** (Table 1, entry 4) and azocarbamate **6** (Table 1, entry 3). The isomerization rates of bis(*tert*-butylcarbonylaminobenzyl)azobenzene **5** could not be measured in toluene and TBME due to the insolubility of the compound in these solvents. In 1,4-dioxane and DMSO the isomerization depends on the temperature. While the rates are in comparison rather long at 25 ºC, these processes are considerably faster at 35 ºC.

**Table 1:** Thermal *Z*→*E* isomerization half-lives τ (standard deviations in parentheses) of azobenzenes **4**–**7** in various solvents.

entry	compound	τ*_Z→E_* [h]							

		toluene		1,4-dioxane		DMSO		TBME	
		25 °C	35 °C	25 °C	35 °C	25 °C	35 °C	25 °C	35 °C

1	**4** R = TMH^a^	97 (6)	32.2 (0.8)	45 (7)	16.7 (0.4)	50.1 (0.2)	14.7 (0.002)	65 (13)	26 (1)
2	**5** R = CO*t*-Bu	–	–	46.3 (0.6)	14.0 (0.1)	50.0 (0.2)	14.4 (0.2)	–	–
3	**6** R = Boc	48 (2)	14.0 (0.6)	39.7 (0.2)	14.9 (0.1)	51.1 (0.3)	14.4 (0.2)	36 (10)	14 (1)
4	**7** R = H	35.4 (0.2)	11.5 (0.5)	38 (2)	12.0 (0.3)	45.7 (0.01)	12.8 (0.3)	37.6 (0.7)	14.3 (0.3)

^a^TMH = 3,5,5-trimethylhexanoyl.

As outlined before, several stabilizing interactions such as LD, hydrogen bonding and solvation effects are possible for the stabilization of *Z*-azobenzenes **4**–**7**. To estimate the influences of those effects, a conformer distribution analysis was performed to identify low-lying conformations of the corresponding (*Z*)-azobenzenes. The energetically favored conformers found (within 1.5 kcal mol^−1^ for **4** and 5 kcal mol^−1^ for **5**–**7**, respectively, relative to the lowest energy conformer) were then re-optimized at the B3LYP/6-31G** [26–29] level of theory with and without D3(BJ) [30–31] dispersion correction (gas phase) (conformations of one enantiomer of each diastereomer of **4** were analyzed. The maximum stabilization was found for (*R*,*S*)-**4**. For the other diastereomer, see Supporting Information File 1).

**Figure 1:**
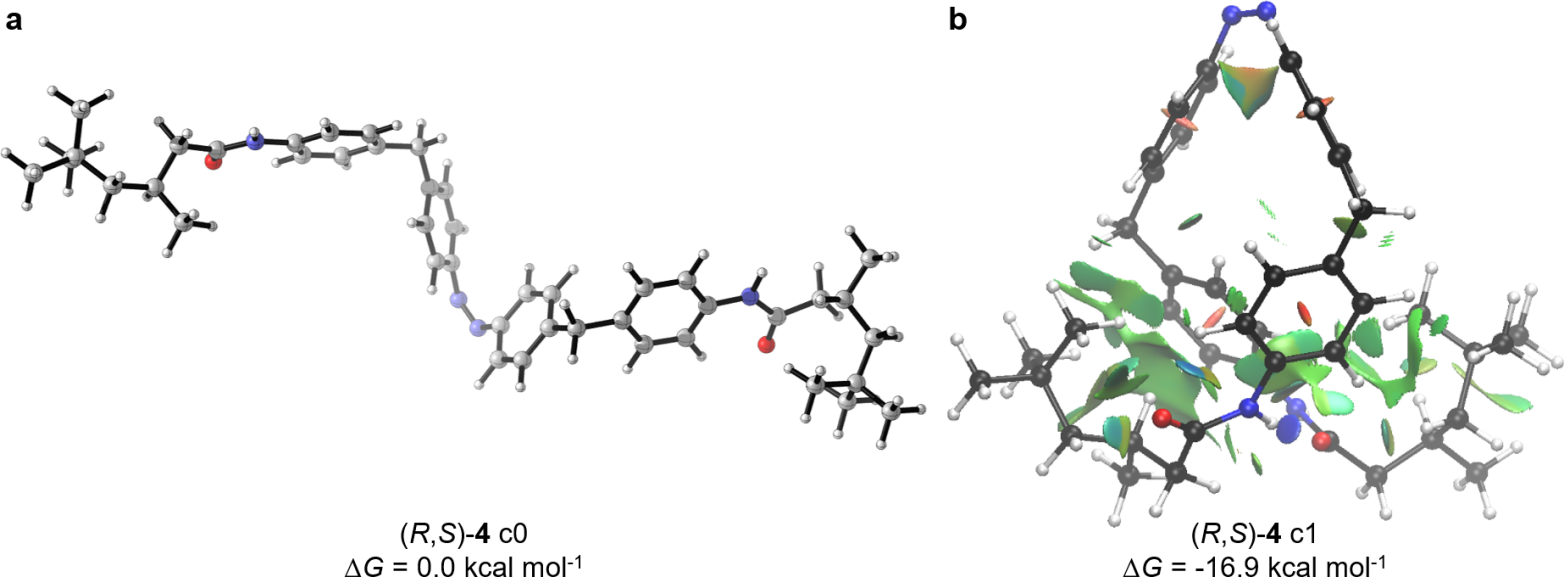
a) Optimized geometry of open conformer (*R*,*S*)-**4** c0. b) NCI plot of the most stable conformer of (*R*,*S*)-**4** (green and blue isosurfaces indicate attractive noncovalent interactions).

As it can be seen in Table 2, the computations reproduced the stabilization of the tight conformations relative to their open forms, which is in agreement with the experimentally observed kinetics of azobenzenes **4**–**7**. Comparing the free energies of azobenzenes **4**–**7** relative to their sterically less crowded conformations, where the diphenylmethane units point away from each other, azodiamide **4** was found to feature the highest stabilization, whereas the *tert*-butylcarbonylamino compound **5** and Boc-protected derivative **6** are almost equally stabilized. Furthermore, azodiamine **7** shows the lowest relative stabilization and thus isomerizes most rapidly. Additionally, all compounds form intramolecular hydrogen bonds between the *N*-substituted moieties. However, the higher relative free energies computed with D3(BJ) dispersion correction, as well as the close H–H contacts in all compounds of about 2.4 Å to 3.0 Å support the stabilizing effect of LD interactions in (*Z*)-azobenzenes **4**–**7**. Figure 1 visualizes the computational findings for the compared conformers of (*Z*)-**4**. A noncovalent interaction (NCI) analysis [32–33] of (*R*,*S*)-**4** in its tightly folded conformation revealed multiple attractive H–H as well as H–π interactions between the amide residues and the aryl groups to be responsible for the overall stabilization (green and blue isosurfaces in Figure 1b).

**Table 2:** Computational results for (*Z*)-azobenzenes **4**–**7**. Δ*G* is the free energy of the most stable *Z*-conformer relative to the corresponding open (*Z*)-conformation (method in parentheses, see Figure S4 in Supporting Information File 1 for graphical representations of the compared conformers).

Compound	**4** R = TMH	**5** R = CO*t*-Bu	**6** R = Boc	**7** R = H

Δ*G* relative to open conformations (B3LYP-D3(BJ)^a^) [kcal mol^−1^]	−16.9	−10.5	−10.7	−3.7
Δ*G* relative to open conformations (B3LYP^a^) [kcal mol^−1^]	1.2	−0.7	0.6	0.5
*d*_NH---X_ [Å]^a,b^	1.87	2.14	1.90	2.11
closest *d*_H---H_ [Å]	2.38	2.53	2.39	–

^a^Basis set: 6-31G**, ^b^X = O for **4**, **5** and **6**; X = N for **7**.

Apparently, hydrogen bonds are much stronger than LD interactions and are mainly responsible for the observed stabilization effects, which is also represented as a blue surface in the NCI plot of (*R*,*S*)-**4** c1. Furthermore, it is known that amides form stronger hydrogen bonds than carbamates and the strength also depends on the steric bulk of the amide [34]. This explains the highest stabilization of the trimethylhexanoylamide **4** (experimentally and computationally), followed by the *tert*-butylamide **5** and *tert*-butyl carbamate **6**, which are almost equally stabilized. Nevertheless, hydrogen bonds are strongly dependent on the solvent system and become weaker with increasing dielectric constant of the solvent [35]. This fact becomes obvious when comparing the half-lives of azobenzene **4** in non-polar toluene or TBME with polar DMSO. In contrast to toluene, the half-life of **4** decreased almost by 50% in DMSO due to the weakening of intramolecular hydrogen bonding. As a result, all azobenzenes despite **7** showed comparable isomerization half-lives in DMSO. Accordingly, LD interactions can be responsible for the slightly but significantly prolonged half-lives compared to azodiamine **7**. Analyzing the isomerization in 1,4-dioxane, weak hydrogen bonds as well as dispersion interactions are operative, which is expressed by the same isomerization half-life trend as in toluene, yet with lower absolute values. These results show, that LD interactions may indeed contribute to the overall stabilization of complex molecules even in the presence of stronger interactions, such as hydrogen bonds [36–37].

## Conclusion

In conclusion, unexpected variations in the isomerization rates of azobenzenes with different remote nitrogen substituents were observed. The experimental and computational investigations reveal a subtle interplay of hydrogen bonding, LD interactions and solvent effects. In general, intramolecular hydrogen bonds were found to have the strongest influence on the observed thermal *Z*→*E* isomerization half-lives. However, LD becomes the decisive factor in polar solvents in which hydrogen bonding plays a minor role. This study demonstrates the importance of even small energy interactions, such as LD, and provides new insights for the application of LD as design element in complex systems in general.

## Experimental

**Synthesis of bis(*tert*-butylcarbonylamino)azobenzene 5**: To a solution of 4,4′-bis(4-aminobenzyl)azobenzene (**7**) [18] (78 mg, 0.20 mmol, 1.0 equiv) and NEt_3_ (65 µL, 0.44 mmol, 2.2 equiv) in THF (2 mL), pivaloyl chloride (54.4 µL, 0.437 mmol, 2.20 equiv) in THF (0.5 mL) was added dropwise at 0 °C. Then, the reaction was allowed to warm to rt while stirring for 2 h. After quenching with sat. aq. NH_4_Cl solution (5 mL), the aqueous phase was extracted with THF (5 mL). The organic phase was washed with sat. aq. NaHCO_3_ (2 × 10 mL) and was dried over MgSO_4_, filtered and concentrated. The residue was suspended in hot EtOH and was filtered while hot. After evaporation of the filtrate, the residue was washed with H_2_O and was dried under high vacuum to yield the product as a yellow solid (39 mg, 35%). ^1^H NMR (400 MHz, DMSO-*d*_6_) δ ppm 9.14 (s, 2H), 7.79 (d, *J* = 8.0 Hz, 4H), 7.56 (d, *J* = 8.2 Hz, 4H), 7.40 (d, *J* = 8.0 Hz, 4H), 7.18 (d, *J* = 8.1 Hz, 4H), 3.98 (s, 4H), 1.20 (s, 18H); ^13^C NMR (101 MHz, DMSO-*d*_6_) δ ppm 176.3, 150.3, 145.3, 137.5, 135.3, 129.6, 128.7, 122.6, 120.5, 40.7*, 39.5*, 27.2; HRMS (ESI) *m*/*z*: [M + Na]^+^ calcd, 583.3043; found, 583.3043. *identified by HSQC and HMBC spectroscopy.

## Supporting Information

File 1 NMR spectra of azobenzene **5**, UV–vis data and detailed procedures, details for conformational analysis and DFT computations.
