# Supplementary material for: London dispersion as important factor for the stabilization of (Z)-azobenzenes in the presence of hydrogen bonding
Source: Beilstein J Org Chem. 2018 May 29;14:1238–43. doi: 10.3762/bjoc.14.106 (PMC6009200; doi:10.3762/bjoc.14.106)
Supplement: File 1 — NMR spectra of azobenzene 5, UV–vis data and detailed procedures, details for conformational analysis and DFT computations. [file Beilstein_J_Org_Chem-14-1238-s001.pdf]

Supporting Information

for

London dispersion as important factor for the stabilization of (*Z*)-azobenzenes in the presence of hydrogen bonding

Andreas H. Heindl,[1] Raffael C. Wende[1] and Hermann A. Wegner*[1]

Address: [1]Institut für Organische Chemie, Justus-Liebig-Universität Gießen, Heinrich-Buff-Ring 17, 35392 Gießen, Germany

Email: Hermann A. Wegner - hermann.a.wegner@org.chemie.uni-giessen.de

* Corresponding author

NMR spectra of azobenzene 5, UV–vis data and detailed procedures, details for conformational analysis and DFT computations

General information S2

NMR spectra S3

Kinetic measurements S4

Computational data S8

Compound (*R*,*S*)-**4** TMH S11

Compound (*S*,*S*)-**4** TMH S25

Compound **5** CO*t*-Bu S39

Compound **6** Boc S51

Compound **7** H S71

References S80

General Information

Chemicals were used as purchased from Sigma-Aldrich, Acros Organics, Alfa Aesar and TCI Europe. Solids were dried over Sicapent® and under high vacuum when necessary. NMR spectra were measured on a Bruker Avance II 200 MHz, Avance II 400 MHz or Avance III 600 MHz spectrometer at room temperature. Chemical shifts are reported in parts per million (ppm) relative to the solvent peak, coupling constants (*J*) are reported in Hertz (Hz). Deuterated solvents were obtained from Deutero GmbH (Kastellaun, Germany). For all azobenzenes, the thermodynamically more stable *E*-isomer is reported. ESI–MS spectra were recorded on a Bruker Daltonics MicrOTOF. Solvents for UV/Vis spectroscopy were purchased from Merck (toluene, TBME, Uvasol®) and Fluka (DMSO) in UV spectroscopic grade. 1,4-Dioxane was purchased from Acros Organics in HPLC quality and was filtered through basic aluminium oxide (Brockmann I, 50-200 µm, 60 A, Acros Organics) before use.

NMR Spectra

Bis-[4,4′-(4-*tert*-butylamidobenzyl)]azobenzene (**5**)

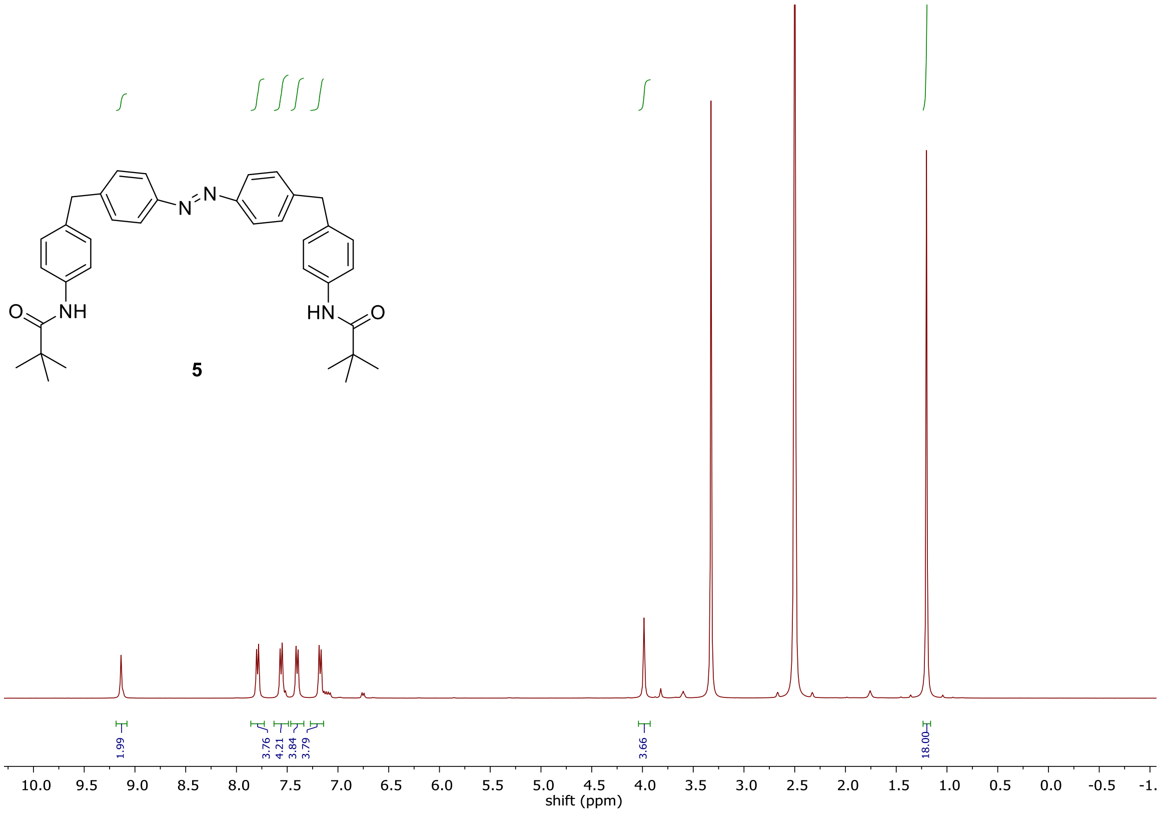

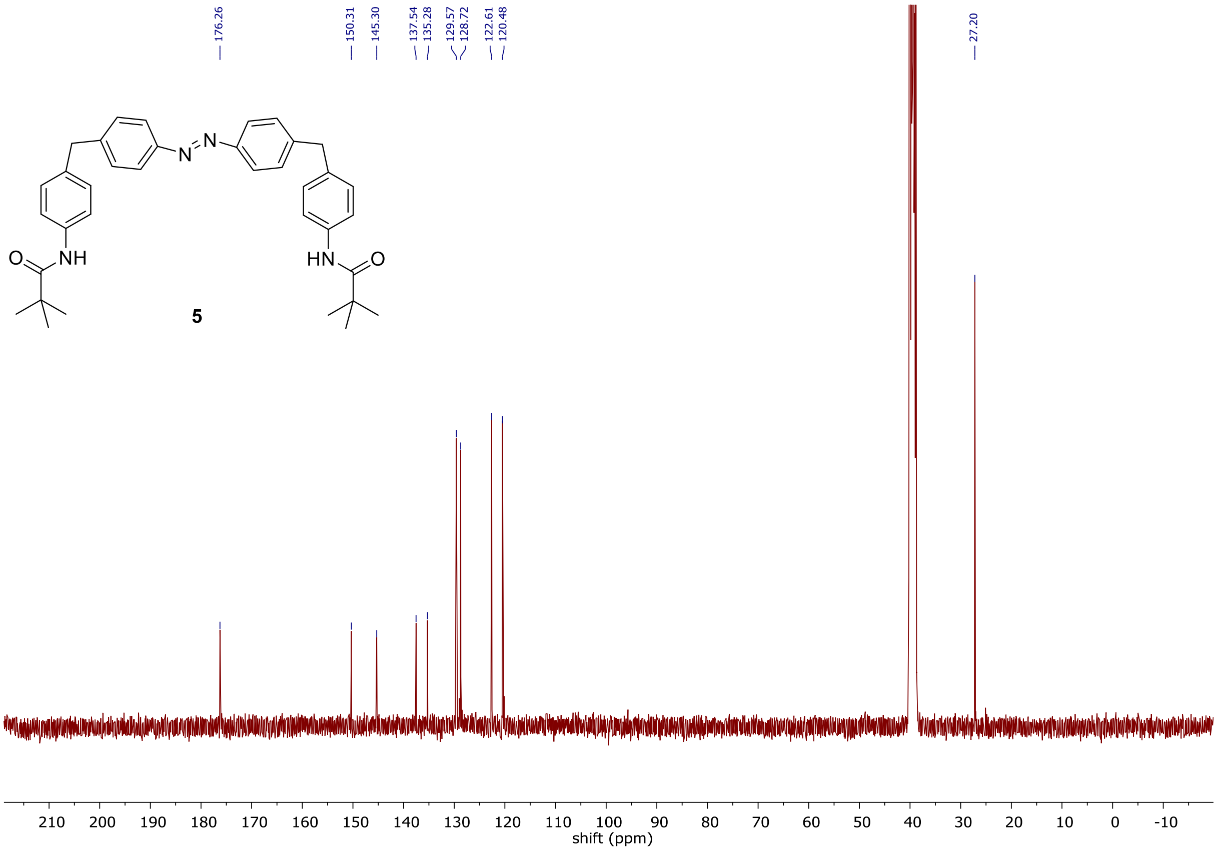

Kinetic measurements

General

The kinetic measurements were carried out with a SPECORD® 200 PLUS spectrophotometer equipped with two automatic eight-fold cell changers and a Peltier element thermostat system (0.1 °C accuracy) by Analytik Jena. The system was operated with the ASpect UV software by Analytik Jena. The sample solutions were measured in QS High Precision Cells made of Quartz Suprasil® with a light path of 10 mm by Hellma Analytics. The sample solution volume was 3 mL with a concentration of $4 \cdot 10^{-5}$ mol/L in all used solvents. Irradiation of the samples for *E*→*Z* isomerization was conducted with a NCSU276AT-U365 UV LED (3 W) at 365 nm (9 nm at FWHM) by Nichia Corporation. All sample solutions of **4**-**7** in the corresponding solvents were irradiated for 14 s and kept in the temperature-controlled spectrometer cells afterwards for approx. 3 min for temperature equilibration before starting the measurement. For each sample, spectra were recorded in one-hour intervals from 285 nm to 650 nm with a scan speed of 20 nm/s. *Z*→*E* isomerizations at 25 °C were monitored for 96 h and at 35 °C for 72 h, resulting in 96 and 72 spectra per run, respectively. For measurements in volatile TBME at 35 °C, the spectra were normalized on the isosbestic points to compensate eventual solvent evaporation.

Half-life calculations

Half-lives were obtained in a double determination at a given temperature. For data analysis, Origin Pro 2016G (64 bit) by Origin Lab Corporation was used. The absorbances A at the maximum wavelengths were plotted against the time t elapsed after the start of the measurements. Rate constants k and half-lives τ were

determined after fitting the data with an Exponential Decay Function (ExpDec1, Origin) according to the literature.[1]

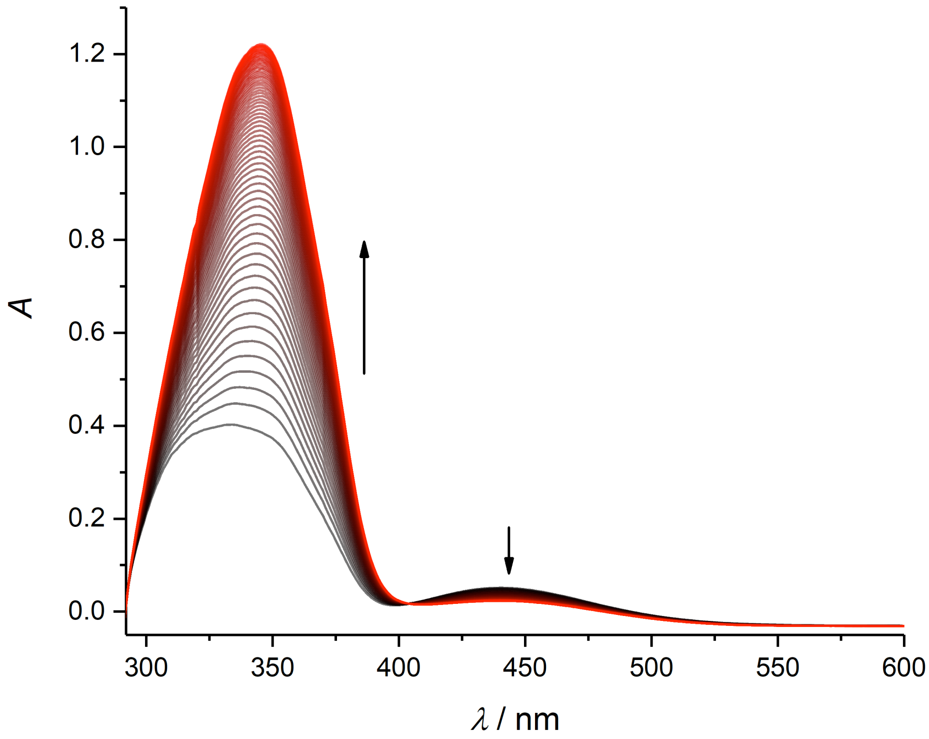

Figure S1: Example reaction spectrum of compound **6** in DMSO at 35 °C in 1 h intervals.

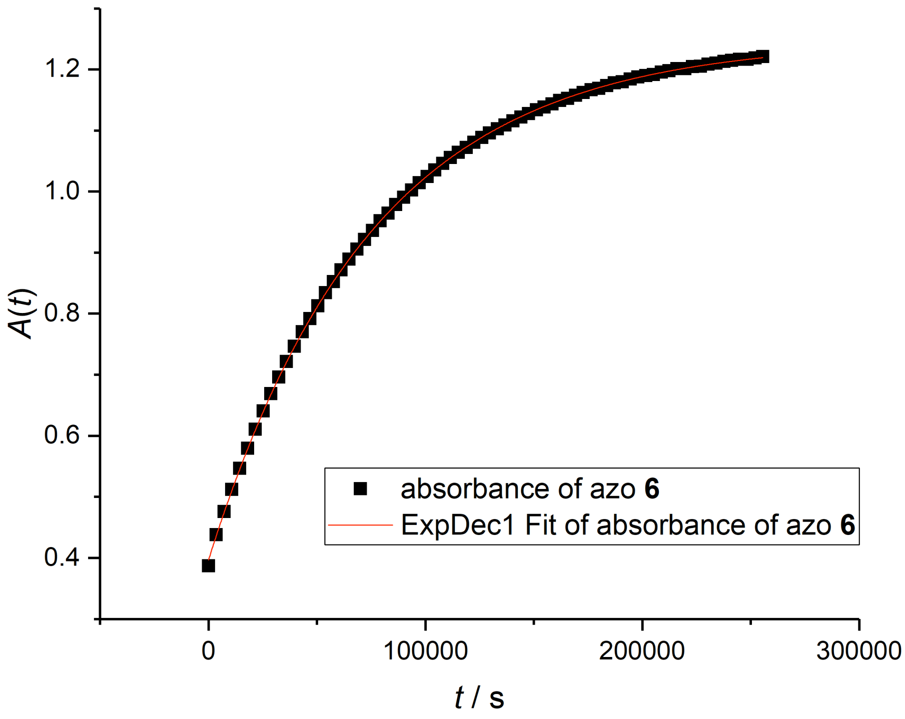

Figure S2: Exponential decay fit of absorbance values as a function of time for azo **6** in DMSO at 35 °C.

Table S1: Kinetic data for azobenzenes **4-7** in DMSO at 25 °C.

**DMSO 25 °C**	k(run 1) / 10^{-6} s^{-1}	k(run 2) / 10^{-6} s^{-1}	average k / 10^{-6} s^{-1}	standard deviation / 10^{-6} s^{-1}	τ (run 1) / h	τ (run 2) / h	average τ / h	standard deviat. / h
**4** TMH	3.851	3.834	3.842	0.012	50.00	50.22	50.11	0.15
**5** CO^tBu	3.862	3.841	3.851	0.015	49.86	50.13	50.00	0.19
**6** Boc	3.758	3.785	3.772	0.019	51.23	50.86	51.05	0.26
**7** H	4.216	4.217	4.217	0.001	45.67	45.65	45.66	0.01

Table S2: Kinetic data for azobenzenes **4-7** in DMSO at 35 °C.

**DMSO 35 °C**	k(run 1) / 10^{-6} s^{-1}	k(run 2) / 10^{-6} s^{-1}	average k / 10^{-6} s^{-1}	standard deviat. / 10^{-6} s^{-1}	τ (run 1) / h	τ (run 2) / h	average τ / h	standard deviat. / h
**4** TMH	13.11	13.11	13.11	0.001	14.69	14.69	14.69	0.002
**5** CO^tBu	13.21	13.47	13.34	0.18	14.57	14.29	14.43	0.20
**6** Boc	13.22	13.47	13.34	0.18	14.57	14.29	14.43	0.19
**7** H	14.84	15.33	15.08	0.34	12.97	12.56	12.77	0.29

Table S3: Kinetic data for azobenzenes **4-7** in toluene at 25 °C.

**toluene 25 °C**	k(run 1) / 10^{-6} s^{-1}	k(run 2) / 10^{-6} s^{-1}	average k / 10^{-6} s^{-1}	standard deviat. / 10^{-6} s^{-1}	τ (run 1) / h	τ (run 2) / h	average τ / h	standard deviat. / h
**4** TMH	1.895	2.077	1.986	0.129	101.63	92.71	97.17	6.31
**5** CO^tBu	insoluble							
**6** Boc	3.921	4.154	4.038	0.165	49.10	46.35	47.73	1.95
**7** H	5.422	5.472	5.447	0.036	35.51	35.18	35.35	0.23

Table S4: Kinetic data for azobenzenes **4-7** in toluene at 35 °C.

**toluene 35 °C**	k(run 1) / 10^{-6} s^{-1}	k(run 2) / 10^{-6} s^{-1}	average k / 10^{-6} s^{-1}	standard deviat. / 10^{-6} s^{-1}	τ (run 1) / h	τ (run 2) / h	average τ / h	standard deviat. / h
**4** TMH	5.880	6.076	5.978	0.139	32.75	31.69	32.22	0.75
**5** CO^tBu	insoluble							
**6** Boc	14.14	13.32	13.73	0.581	13.62	14.46	14.04	0.59
**7** H	17.34	16.25	16.79	0.765	11.11	11.85	11.48	0.52

Table S5: Kinetic data for azobenzenes **4-7** in 1,4-dioxane at 25 °C.

**dioxane 25 °C**	*k*(run 1) / 10^{-6} s^{-1}	*k*(run 2) / 10^{-6} s^{-1}	average *k* / 10^{-6} s^{-1}	standard deviat. / 10^{-6} s^{-1}	τ (run 1) / h	τ (run 2) / h	average τ / h	standard deviat. / h
**4** TMH	3.889	4.770	4.329	0.622	49.50	40.37	44.94	6.46
**5** CO^{t}Bu	4.119	4.195	4.157	0.053	46.74	45.90	46.32	0.59
**6** Boc	4.841	4.872	4.856	0.022	39.78	39.52	39.65	0.18
**7** H	4.976	5.288	5.132	0.221	38.69	36.41	37.55	1.61

Table S6: Kinetic data for azobenzenes **4-7** in 1,4-dioxane at 35 °C.

**dioxane 35 °C**	*k*(run 1) / 10^{-6} s^{-1}	*k*(run 2) / 10^{-6} s^{-1}	average *k* / 10^{-6} s^{-1}	standard deviat. / 10^{-6} s^{-1}	τ (run 1) / h	τ (run 2) / h	average τ / h	standard deviat. / h
**4** TMH	11.70	11.34	11.52	0.255	16.46	16.98	16.72	0.37
**5** CO^{t}Bu	12.95	12.87	12.91	0.053	14.87	14.95	14.91	0.06
**6** Boc	13.67	13.77	13.72	0.078	14.08	13.98	14.03	0.07
**7** H	16.25	15.74	15.99	0.361	11.85	12.23	12.04	0.27

Table S7: Kinetic data for azobenzenes **4-7** in TBME at 25 °C.

**TBME 25 °C**	*k*(run 1) / 10^{-6} s^{-1}	*k*(run 2) / 10^{-6} s^{-1}	average *k* / 10^{-6} s^{-1}	standard deviat. / 10^{-6} s^{-1}	τ (run 1) / h	τ (run 2) / h	average τ / h	standard deviat. / h
**4** TMH	2.584	3.441	3.012	0.606	74.52	55.96	65.24	13.13
**5** CO^{t}Bu	insoluble							
**6** Boc	6.716	4.441	5.578	1.609	28.67	43.36	36.02	10.39
**7** H	5.052	5.184	5.118	0.094	38.11	37.14	37.63	0.69

Table S8: Kinetic data for azobenzenes **4-7** in TBME at 35 °C.

**TBME 35 °C**	*k*(run 1) / 10^{-6} s^{-1}	*k*(run 2) / 10^{-6} s^{-1}	average *k* / 10^{-6} s^{-1}	standard deviat. / 10^{-6} s^{-1}	τ (run 1) / h	τ (run 2) / h	average τ / h	standard deviat. / h
**4** TMH	7.743	7.090	7.416	0.461	24.87	27.16	26.01	1.62
**5** CO^{t}Bu	insoluble							
**6** Boc	14.72	12.68	13.70	1.446	13.08	15.19	14.13	1.49
**7** H	13.21	13.66	13.43	0.318	14.58	14.10	14.34	0.34

Computational Data

Prior to the DFT geometry optimization, a conformer distribution analysis was performed to identify low-lying conformations of azo-derivatives **4**-**7** with Spartan '16, Version 2.0.3, employing the Merck molecular force field (MMFF).[2] The energetically favored conformers (within 1.5 kcal mol^{-1} for **4** and 5 kcal mol^{-1} for **5**-**7**, respectively, relative to the lowest-lying conformer) found were then re-optimized on the B3LYP and B3LYP-D3(BJ) level of theory using a 6-31G** basis set.[3–8] In addition, the opt=tight and int=ultrafine keywords were applied. The absence of imaginary frequencies in ground state computations was checked for every optimized geometry. Results of the NCI analysis were visualized using VMD.[9]

Optimized Geometries

All obtained geometries from the conformer analysis were optimized by DFT computations. In some cases, different starting conformers converged to the same minimum energy geometry. Therefore, only optimized geometries of different energy are given for the investigated compounds **4**-**7**. Structures were visualized with CYLview.[10]

Table S9: Relative free energies of DFT-optimized geometries of conformations of azo **4**.

**4** TMH	Δ*G* B3LYP-D3(BJ) / kcal mol^{-1}	Δ*G* B3LYP / kcal mol^{-1}
(*R*,*S*)-c0	0.0	0.0
(*R*,*S*)-c1	-16.9	3.99
(*R*,*S*)-c2	-16.6	1.24
(*S*,*S*)-c0	0.0	0.0
(*S*,*S*)-c1	-11.6	-3.1
(*S*,*S*)-c2	-12.7	-3.1

Table S10: Relative free energies of DFT-optimized geometries of conformations of azo **5**.

**5** CO^{t}Bu	ΔG B3LYP-D3(BJ) / kcal mol^{-1}	ΔG B3LYP / kcal mol^{-1}
c0	0.0	0.0
c1	-10.5	-0.7
c2	-1.7	-0.7

Table S11: Relative free energies of DFT-optimized geometries of conformations of azo **6**.

**6** Boc	ΔG B3LYP-D3(BJ) / kcal mol^{-1}	ΔG B3LYP / kcal mol^{-1}
c0	0.0	0.0
c1	-10.7	0.6
c2	-7.4	2.3
c3	-7.5	2.1
c4	-7.8	2.1

Table S12: Relative free energies of DFT-optimized geometries of conformations of azo **7**.

**7** H	ΔG B3LYP-D3(BJ) / kcal mol^{-1}	ΔG B3LYP / kcal mol^{-1}
c0	0.0	0.0
c1	-3.7	0.5
c2	0.0	-0.1

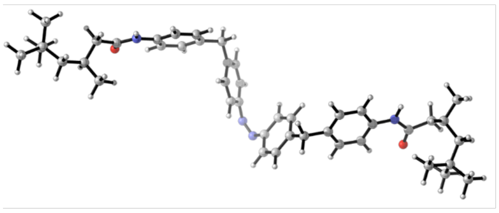

(R,S)-**4** c0
ΔG = 0.0 kcal mol^{-1}

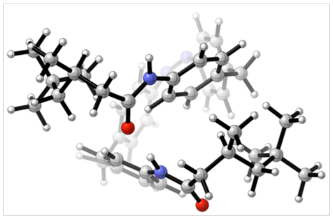

(R,S)-**4** c1
ΔG = -16.9 kcal mol^{-1}

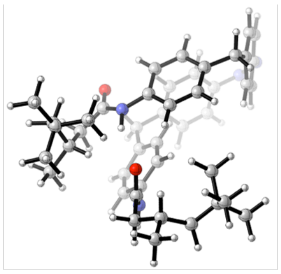

(R,S)-**4** c2
ΔG = -16.6 kcal mol^{-1}

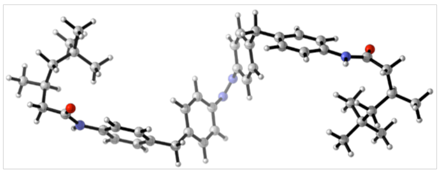

(S,S)-**4** c0
ΔG = 0.0 kcal mol^{-1}

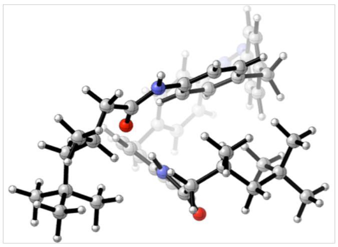

(S,S)-**4** c1
ΔG = -11.6 kcal mol^{-1}

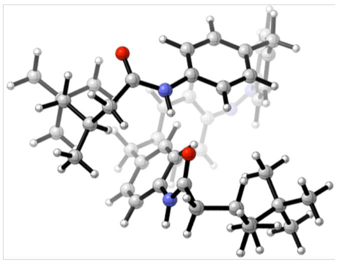

(S,S)-**4** c2
ΔG = -12.7 kcal mol^{-1}

B3LYP-D3(BJ)/6-31G**

Figure S3: Conformers of (R,S) and (S,S)-azo **4** with their free energies relative to their open conformations.

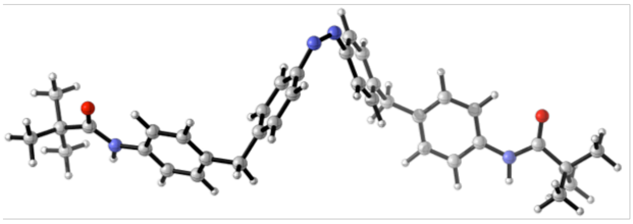

5 c0
Δ*G* = 0.0 kcal mol^{-1}

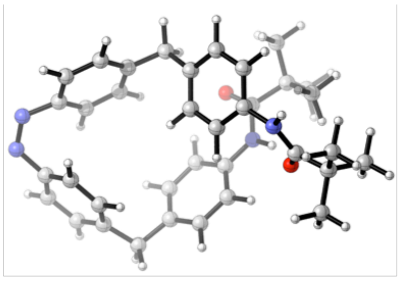

5 c1
Δ*G* = -10.5 kcal mol^{-1}

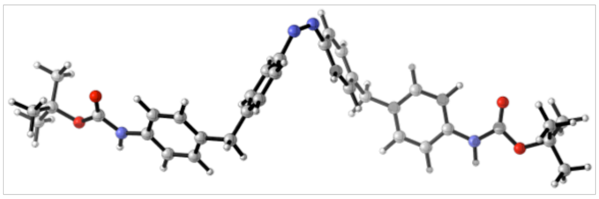

6 c0
Δ*G* = 0.0 kcal mol^{-1}

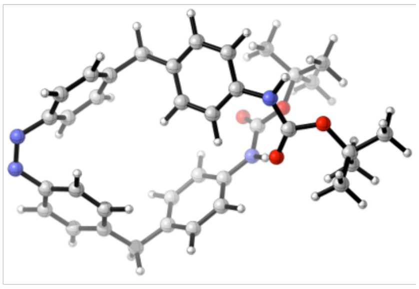

6 c1
Δ*G* = -10.7 kcal mol^{-1}

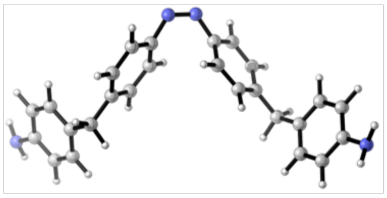

7 c0
Δ*G* = 0.0 kcal mol^{-1}

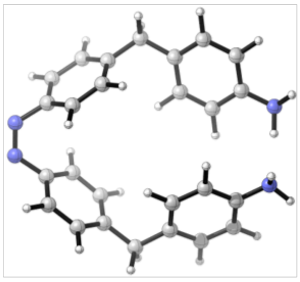

7 c1
Δ*G* = -3.7 kcal mol^{-1}

Figure S4: Open conformations of azobenzenes **5**-**7** c0 (left) and their corresponding most stabilized conformers **5**-**7** c1 (right, B3LYP-D3(BJ)/6-31G**).

Compound (*R*,*S*)-4 TMH
Conformer 0 without dispersion
C -2.365691 -0.086146 0.911179
C -2.561623 -1.230597 1.699873
C -2.010660 -1.248392 2.986879
C -1.289995 -0.160902 3.476041
C -1.052421 0.949044 2.657946
C -1.613638 0.988463 1.371369
N -0.409460 2.074226 3.272709
N 0.465701 2.772309 2.715539
C 1.099554 2.428670 1.475571
C 1.652058 1.164241 1.215136
C 2.396430 0.959050 0.059340
C 2.592969 1.988542 -0.874365

C 2.050574 3.248252 -0.592989
C 1.337925 3.478531 0.581911
C -3.345644 -2.419219 1.168390
C -4.725488 -2.083231 0.630019
C 3.369137 1.740057 -2.157146
C 4.750201 1.139370 -1.960180
C -5.079354 -2.373920 -0.691338
C -6.351382 -2.082405 -1.177325
C -7.311711 -1.487439 -0.347862
C -6.972555 -1.186905 0.981786
C -5.694755 -1.484752 1.446394
C 5.103093 -0.077619 -2.551876
C 6.376485 -0.616534 -2.386768
C 7.339031 0.052571 -1.618614
C 7.000372 1.274552 -1.014184
C 5.721558 1.794752 -1.191386
N -8.580126 -1.221053 -0.906283
C -9.687514 -0.648686 -0.320288
C -10.876310 -0.456216 -1.260120
C -12.212921 -0.856263 -0.600324
C -13.430116 -0.288319 -1.374279
C -14.049918 1.041218 -0.859094
O -9.733021 -0.291143 0.850429
C -12.300661 -2.384238 -0.461097
C -14.679560 0.831195 0.532621
C -13.004999 2.171031 -0.778456
C -15.159456 1.458450 -1.844764
N 8.608688 -0.550995 -1.494627
C 9.727808 -0.098228 -0.831353
O 9.767411 0.946163 -0.192520
C 10.957711 -0.992693 -0.977064
C 11.719679 -1.161106 0.354203
C 10.923107 -2.060334 1.312631
H 9.908151 -1.680298 1.469647
C 13.154920 -1.702868 0.131502
H -2.815911 -0.037040 -0.076442
H -2.164734 -2.116303 3.622993
H -0.899982 -0.158312 4.489087
H -1.473716 1.863556 0.746533
H 1.511510 0.355857 1.924031
H 2.840097 -0.015971 -0.122378
H 2.205209 4.065838 -1.292477
H 0.954732 4.466021 0.819404
H -3.435305 -3.163918 1.969021
H -2.767104 -2.904135 0.371375
H 3.455762 2.688614 -2.701587
H 2.786690 1.073724 -2.806606
H -4.353541 -2.839313 -1.353510
H -6.600265 -2.319169 -2.209658
H -7.709236 -0.728532 1.625614
H -5.444933 -1.238724 2.475172

H 4.375606 -0.613941 -3.156021
H 6.624809 -1.564736 -2.858966
H 7.738012 1.793575 -0.419364
H 5.472332 2.737324 -0.711017
H -8.681082 -1.482559 -1.876177
H -10.743523 -1.008297 -2.200371
H -10.891938 0.607789 -1.520994
H -12.191942 -0.434048 0.409837
H -14.232076 -1.038152 -1.368314
H -13.157976 -0.161791 -2.432142
H -12.337467 -2.869398 -1.444743
H -13.203711 -2.675756 0.084669
H -11.440387 -2.788371 0.082869
H -13.934876 0.539998 1.280163
H -15.449494 0.051594 0.503443
H -15.153281 1.753823 0.885988
H -12.207360 1.941697 -0.064655
H -13.476307 3.104963 -0.452050
H -12.546067 2.361049 -1.756061
H -15.926138 0.680061 -1.932427
H -14.753059 1.641328 -2.846191
H -15.654724 2.377822 -1.512886
H 8.709205 -1.432567 -1.976343
H 11.603165 -0.508736 -1.718505
H 10.695328 -1.978191 -1.385091
H 11.770425 -0.164078 0.804102
H 11.410167 -2.116565 2.291377
H 10.845208 -3.083096 0.922506
H 13.176050 -2.300426 -0.791349
H 13.394768 -2.406963 0.939024
C 14.311734 -0.665355 0.077490
C 14.069040 0.413853 -0.995541
H 13.173763 1.007628 -0.785108
H 14.917051 1.106596 -1.039235
H 13.959093 -0.031003 -1.991767
C 14.482392 0.013605 1.451292
H 14.686248 -0.726108 2.234012
H 15.321086 0.718446 1.432848
H 13.590248 0.575798 1.744838
C 15.610458 -1.422877 -0.263306
H 15.821165 -2.202998 0.477210
H 15.543443 -1.904217 -1.245793
H 16.468905 -0.742171 -0.283311

E = -2079.97273904 Hartree
ZPVE = 574.45286 kcal/mol
Zero-point correction= 0.915449 (Hartree/Particle)
Thermal correction to Energy= 0.966452
Thermal correction to Enthalpy= 0.967397
Thermal correction to Gibbs Free Energy= 0.815553
Sum of electronic and zero-point Energies= -2079.057290

Sum of electronic and thermal Energies= -2079.006287
Sum of electronic and thermal Enthalpies= -2079.005342
Sum of electronic and thermal Free Energies= -2079.157186

Conformer 0 with dispersion
C 2.359645 0.991538 -0.663200
C 2.555146 0.101612 -1.729875
C 2.023129 0.433570 -2.979396
C 1.312392 1.617497 -3.163993
C 1.072511 2.464026 -2.078915
C 1.619382 2.154625 -0.824133
N 0.412858 3.704696 -2.360059
N -0.478082 4.185206 -1.625083
C -1.069003 3.461332 -0.538716
C -1.559184 2.152741 -0.667236
C -2.237823 1.564319 0.391029
C -2.425724 2.246424 1.602382
C -1.951400 3.557294 1.708358
C -1.304057 4.173707 0.639788
C 3.335348 -1.181841 -1.526787
C 4.679109 -0.978737 -0.857653
C -3.136516 1.574714 2.760545
C -4.460986 0.946494 2.379335
C 5.021437 -1.665193 0.309651
C 6.260928 -1.478250 0.914371
C 7.193374 -0.590454 0.363279
C 6.862492 0.112473 -0.806071
C 5.618412 -0.089653 -1.393924
C -4.716951 -0.407300 2.608885
C -5.937923 -0.975922 2.257925
C -6.938357 -0.197851 1.662151
C -6.695431 1.163654 1.420256
C -5.468012 1.711043 1.777699
N 8.431230 -0.457826 1.022576
C 9.520050 0.313454 0.681364
C 10.713184 0.128650 1.612349
C 11.259138 -1.320885 1.743649
C 12.460221 -1.649944 0.833920
C 12.312058 -1.659995 -0.711923
O 9.526967 1.089111 -0.266266
C 11.658357 -1.589654 3.203603
C 11.004323 -2.344351 -1.144394
C 12.388976 -0.244131 -1.312224
C 13.499013 -2.473585 -1.265349
N -8.148560 -0.837763 1.329255
C -9.271673 -0.310254 0.735811
O -9.385123 0.861966 0.399953
C -10.395327 -1.308751 0.497878
C -10.741509 -1.395878 -1.001028
C -9.513507 -1.853631 -1.801780
H -8.668129 -1.173618 -1.663518

C -11.932338 -2.318880 -1.317666
H 2.803093 0.766808 0.302222
H 2.183836 -0.232525 -3.822624
H 0.928963 1.895947 -4.140097
H 1.469486 2.828841 0.010933
H -1.414200 1.610843 -1.594440
H -2.638316 0.561055 0.280397
H -2.107973 4.109211 2.631076
H -0.966527 5.202812 0.705729
H 3.474180 -1.668145 -2.499987
H 2.740282 -1.878183 -0.922540
H -3.290082 2.313309 3.556552
H -2.486383 0.800373 3.186984
H 4.312070 -2.358279 0.753686
H 6.506832 -2.024371 1.822164
H 7.577153 0.802703 -1.228678
H 5.366295 0.468666 -2.291031
H -3.953542 -1.028366 3.069587
H -6.115362 -2.032426 2.445142
H -7.465103 1.763351 0.957527
H -5.283732 2.762080 1.573839
H 8.540665 -1.041639 1.839025
H 10.394756 0.478923 2.603136
H 11.491163 0.813393 1.272182
H 10.453252 -2.021534 1.490807
H 12.789061 -2.654724 1.130408
H 13.288397 -0.977260 1.098229
H 12.456201 -0.907947 3.519870
H 12.024020 -2.612781 3.334226
H 10.811131 -1.444564 3.883112
H 10.121583 -1.763224 -0.866274
H 10.910962 -3.341245 -0.697622
H 10.981393 -2.465380 -2.232435
H 11.546762 0.387074 -1.027005
H 12.393769 -0.302511 -2.406490
H 13.316590 0.253664 -1.006377
H 13.463262 -3.514043 -0.923273
H 14.454467 -2.045927 -0.941110
H 13.489905 -2.477976 -2.360304
H -8.184846 -1.823041 1.543634
H -11.253149 -0.936267 1.061185
H -10.155309 -2.306322 0.887814
H -10.986224 -0.377325 -1.320522
H -9.736955 -1.892055 -2.872255
H -9.196038 -2.857768 -1.493831
H -11.678820 -3.346010 -1.017093
H -12.007546 -2.344433 -2.412550
C -13.347948 -1.981107 -0.770984
C -13.546416 -2.470840 0.676639
H -12.909866 -1.948628 1.393654
H -14.583976 -2.313715 0.990950

H -13.333162 -3.542447 0.758119
C -13.631938 -0.472455 -0.857421
H -13.493759 -0.103302 -1.879571
H -14.665515 -0.260334 -0.563643
H -12.975901 0.108801 -0.202681
C -14.366173 -2.729058 -1.654181
H -14.319958 -2.380491 -2.691438
H -14.170579 -3.807361 -1.653488
H -15.388013 -2.573903 -1.291719

E = -2080.19491537 Hartree
ZPVE = 575.83944 kcal/mol
Zero-point correction= 0.917659 (Hartree/Particle)
Thermal correction to Energy= 0.968319
Thermal correction to Enthalpy= 0.969263
Thermal correction to Gibbs Free Energy= 0.819391
Sum of electronic and zero-point Energies= -2079.277257
Sum of electronic and thermal Energies= -2079.226597
Sum of electronic and thermal Enthalpies= -2079.225653
Sum of electronic and thermal Free Energies= -2079.375524

Conformer 1 without dispersion
N 6.855131 -1.800569 -0.905241
N 6.613538 -1.211973 -1.982610
C 5.880255 -1.950141 0.137138
C 4.111342 -2.388918 2.285792
C 4.587702 -2.442510 -0.101063
C 6.305759 -1.745711 1.453267
C 5.415980 -1.934473 2.510729
C 3.725714 -2.663359 0.964541
H 4.266789 -2.652709 -1.115077
H 7.324750 -1.416781 1.632333
H 5.745973 -1.735461 3.527067
H 2.723837 -3.033246 0.766628
C 5.416784 -0.449714 -2.209520
C 3.207125 1.165013 -2.879256
C 4.742724 -0.642252 -3.422388
C 5.019389 0.588404 -1.357352
C 3.934154 1.390000 -1.702866
C 3.631253 0.135630 -3.732425
H 5.092031 -1.413244 -4.102249
H 5.563240 0.771546 -0.437268
H 3.643772 2.202970 -1.042270
H 3.094516 -0.045441 -4.660203
C 3.121919 -2.570578 3.428792
H 2.805587 -3.620213 3.473159
H 3.627454 -2.356798 4.376712
C 1.997636 2.012596 -3.219795
H 1.997501 2.238201 -4.292311
H 2.089168 2.979577 -2.707737
C 1.897364 -1.684474 3.278305

C -0.308120 0.029654 2.818654
C 1.931936 -0.335090 3.650775
C 0.718234 -2.167655 2.699234
C -0.368500 -1.329204 2.468241
C 0.853436 0.520422 3.438024
H 2.831119 0.066255 4.112333
H 0.644503 -3.216689 2.422075
H -1.273514 -1.721226 2.012939
H 0.894460 1.557750 3.737556
C 0.650615 1.399448 -2.853876
C -1.896711 0.404915 -2.133621
C -0.453044 1.562830 -3.699648
C 0.451503 0.709460 -1.651592
C -0.798319 0.212404 -1.284551
C -1.709509 1.078299 -3.348273
H -0.333990 2.085544 -4.645172
H 1.289852 0.544672 -0.981853
H -0.919450 -0.317745 -0.351661
H -2.551539 1.226895 -4.020858
N -1.425889 0.826707 2.504086
H -2.105487 0.384541 1.892597
N -3.200341 -0.070408 -1.846669
H -3.842488 -0.033266 -2.625682
C -1.668769 2.135195 2.846708
C -3.738867 -0.478122 -0.655447
O -3.107214 -0.514028 0.403128
O -0.924850 2.811057 3.554240
C -2.943490 2.705360 2.233301
H -3.634241 1.895603 1.973185
H -3.425979 3.322188 2.999075
C -2.670933 3.569242 0.970558
H -2.046599 2.964310 0.300394
C -1.922806 4.882980 1.323577
H -1.493916 4.786007 2.325673
H -2.661384 5.695440 1.381504
C -0.773147 5.333145 0.379838
C -0.266746 6.699626 0.884096
H 0.565104 7.062563 0.269481
H 0.086322 6.631941 1.918994
H -1.060876 7.454527 0.850237
C -1.246827 5.487094 -1.078002
H -0.431620 5.858336 -1.710336
H -2.075178 6.201091 -1.155585
H -1.578331 4.533584 -1.502583
C 0.392825 4.325093 0.436588
H 1.244666 4.690536 -0.149898
H 0.110859 3.348481 0.029528
H 0.728772 4.170873 1.466964
C -5.187619 -0.936209 -0.745380
H -5.647352 -0.733441 0.226441
H -5.724835 -0.340354 -1.494121

C -5.340061 -2.444958 -1.089700
H -4.760664 -2.625353 -2.005051
C -4.790647 -3.349784 0.045619
H -4.152788 -2.751383 0.704906
H -5.637945 -3.677561 0.663579
C -3.970183 -4.603368 -0.368586
C -4.764189 -5.525353 -1.313478
H -5.706634 -5.848646 -0.856970
H -4.185028 -6.425458 -1.548286
H -4.999318 -5.034359 -2.263798
C -3.632731 -5.383844 0.917729
H -3.065680 -4.763383 1.620715
H -4.542325 -5.720179 1.428040
H -3.029289 -6.270098 0.692185
C -2.651883 -4.184108 -1.049641
H -2.061058 -3.530862 -0.398344
H -2.823713 -3.648570 -1.989524
H -2.042394 -5.063385 -1.285842
C -4.001491 3.839784 0.249128
H -4.485600 2.906565 -0.061378
H -3.854854 4.454739 -0.643163
H -4.697927 4.373301 0.907320
C -6.816606 -2.736945 -1.403130
H -7.174309 -2.140494 -2.250037
H -7.448808 -2.502257 -0.538761
H -6.970431 -3.790891 -1.647755

E = -2079.98117444 Hartree
ZPVE = 575.32179 kcal/mol
Zero-point correction= 0.916834 (Hartree/Particle)
Thermal correction to Energy= 0.967152
Thermal correction to Enthalpy= 0.968096
Thermal correction to Gibbs Free Energy= 0.825959
Sum of electronic and zero-point Energies= -2079.064341
Sum of electronic and thermal Energies= -2079.014022
Sum of electronic and thermal Enthalpies= -2079.013078
Sum of electronic and thermal Free Energies= -2079.155216

Conformer 1 with dispersion
N 6.292121 -1.960680 -1.215102
N 6.084165 -1.135702 -2.133178
C 5.333131 -2.200151 -0.176775
C 3.567345 -2.753128 1.935259
C 3.982491 -2.471854 -0.442405
C 5.809917 -2.275685 1.132814
C 4.923115 -2.520419 2.180723
C 3.118259 -2.748660 0.605834
H 3.617877 -2.450367 -1.462589
H 6.865757 -2.107459 1.318386
H 5.291355 -2.534388 3.202774
H 2.067844 -2.931339 0.399280

C 4.960185 -0.243134 -2.122161
C 2.846555 1.598125 -2.287029
C 4.234988 -0.110523 -3.311127
C 4.664194 0.583801 -1.032062
C 3.622023 1.501023 -1.125750
C 3.167980 0.778499 -3.376343
H 4.507078 -0.725608 -4.162712
H 5.244195 0.504308 -0.120168
H 3.397805 2.144765 -0.279753
H 2.581180 0.849602 -4.288058
C 2.580908 -2.969775 3.068153
H 2.182415 -3.990580 3.025413
H 3.105748 -2.877294 4.025078
C 1.667942 2.541410 -2.355875
H 1.653164 3.051905 -3.324684
H 1.784977 3.322038 -1.597621
C 1.437530 -1.980580 2.989216
C -0.608407 -0.072603 2.619081
C 1.648747 -0.624987 3.269770
C 0.171139 -2.368741 2.545197
C -0.843173 -1.432858 2.365406
C 0.649446 0.327286 3.098553
H 2.628424 -0.302547 3.612998
H -0.025839 -3.415419 2.328660
H -1.824774 -1.743675 2.023089
H 0.820151 1.369908 3.324770
C 0.332481 1.849611 -2.148705
C -2.160086 0.598091 -1.758227
C -0.755744 2.142795 -2.976292
C 0.144730 0.918923 -1.118477
C -1.081264 0.291691 -0.921303
C -1.992280 1.533353 -2.782805
H -0.637395 2.862013 -3.781519
H 0.973333 0.658185 -0.467718
H -1.190262 -0.438015 -0.134219
H -2.828368 1.779878 -3.431702
N -1.643143 0.823270 2.313089
H -2.370654 0.440549 1.714826
N -3.410063 -0.063245 -1.658586
H -3.930546 -0.140182 -2.520423
C -1.730392 2.159512 2.603261
C -3.997128 -0.604438 -0.550630
O -3.537789 -0.497289 0.588053
O -0.946491 2.760381 3.334714
C -2.858842 2.856464 1.858282
H -3.582066 2.122612 1.488692
H -3.379338 3.522716 2.553678
C -2.294927 3.679235 0.671185
H -1.579084 3.034521 0.148540
C -1.569978 4.952911 1.173569
H -1.407942 4.866196 2.251151

H -2.234428 5.815163 1.023065
C -0.197552 5.274959 0.535790
C 0.290659 6.612851 1.117266
H 1.272251 6.882647 0.711691
H 0.381023 6.556431 2.207264
H -0.405584 7.424681 0.878421
C -0.307751 5.406056 -0.991931
H 0.662592 5.667847 -1.429682
H -1.018318 6.193371 -1.268389
H -0.640081 4.473028 -1.454586
C 0.823486 4.180942 0.899168
H 1.815727 4.439042 0.509804
H 0.541257 3.208409 0.486353
H 0.896466 4.062776 1.983208
C -5.232062 -1.435001 -0.835680
H -5.841175 -1.439345 0.071754
H -5.824722 -0.979202 -1.638396
C -4.858028 -2.887277 -1.231941
H -4.184542 -2.814861 -2.095152
C -4.124458 -3.625116 -0.083590
H -3.862318 -2.910523 0.702616
H -4.823178 -4.336355 0.375421
C -2.834189 -4.391031 -0.469447
C -3.109573 -5.419111 -1.579007
H -3.887119 -6.128992 -1.276030
H -2.204435 -5.992294 -1.806246
H -3.433552 -4.939085 -2.508113
C -2.334528 -5.129195 0.784792
H -2.159441 -4.428790 1.608686
H -3.065802 -5.869906 1.126147
H -1.394585 -5.654143 0.582650
C -1.739568 -3.412087 -0.929556
H -1.471914 -2.719874 -0.127247
H -2.049558 -2.817824 -1.794888
H -0.832867 -3.955870 -1.215824
C -3.420517 4.004025 -0.317786
H -3.870592 3.088894 -0.717401
H -3.046885 4.590505 -1.161708
H -4.210877 4.586763 0.170144
C -6.116725 -3.637033 -1.683698
H -6.597075 -3.143316 -2.535496
H -6.847717 -3.689392 -0.869037
H -5.875363 -4.661384 -1.979216

E = -2080.23873454 Hartree
ZPVE = 577.53449 kcal/mol
Zero-point correction= 0.920360 (Hartree/Particle)
Thermal correction to Energy= 0.969579
Thermal correction to Enthalpy= 0.970523
Thermal correction to Gibbs Free Energy= 0.836356
Sum of electronic and zero-point Energies= -2079.318375

Sum of electronic and thermal Energies= -2079.269155
Sum of electronic and thermal Enthalpies= -2079.268211
Sum of electronic and thermal Free Energies= -2079.402378

Conformer 2 without dispersion
N -7.938655 -1.121175 0.112748
N -7.567450 -2.084996 0.817655
C -7.043126 -0.136056 -0.424168
C -5.561617 2.002211 -1.510499
C -7.244622 0.260731 -1.750818
C -6.127091 0.566750 0.372628
C -5.407842 1.627750 -0.167687
C -6.485465 1.295443 -2.291632
H -7.986553 -0.259023 -2.349095
H -5.991387 0.288473 1.411958
H -4.705344 2.167972 0.460314
H -6.629365 1.573548 -3.332726
C -6.192859 -2.452860 0.996761
C -3.594192 -3.444452 1.453621
C -5.302617 -2.623237 -0.074644
C -5.796620 -2.827114 2.285470
C -4.499932 -3.283961 2.510288
C -4.026200 -3.122359 0.157930
H -5.616595 -2.377643 -1.083075
H -6.507569 -2.737289 3.101036
H -4.192092 -3.538840 3.521288
H -3.343420 -3.251523 -0.676658
C -4.804815 3.184215 -2.095721
H -4.862751 3.124608 -3.190214
H -5.325324 4.112329 -1.824211
C -2.198883 -3.996451 1.699410
H -2.184438 -5.062638 1.437143
H -1.994306 -3.954201 2.777145
C -3.350900 3.308475 -1.678052
C -0.628101 3.562757 -0.962880
C -2.449670 2.248974 -1.849153
C -2.856767 4.500028 -1.136297
C -1.516452 4.630696 -0.783583
C -1.105114 2.358762 -1.502989
H -2.805466 1.308146 -2.259518
H -3.527317 5.342316 -0.987744
H -1.159761 5.567553 -0.361130
H -0.429644 1.528297 -1.645323
C -1.090215 -3.294935 0.935905
C 0.987949 -1.991340 -0.478872
C -0.903800 -1.908907 1.041929
C -0.214375 -4.003835 0.108807
C 0.817242 -3.378546 -0.593688
C 0.112400 -1.264443 0.348664
H -1.572709 -1.323129 1.667040
H -0.335262 -5.079683 0.004472

H 1.480972 -3.943701 -1.231582
H 0.232240 -0.188605 0.433451
N 0.723305 3.778226 -0.597023
H 0.945158 4.730664 -0.345283
N 1.988798 -1.262877 -1.153038
H 1.980350 -0.261482 -0.983239
C 1.758357 2.888865 -0.513718
C 2.931961 -1.725982 -2.038335
O 3.045983 -2.904069 -2.370948
O 1.637530 1.685249 -0.751146
C 3.100752 3.487415 -0.115517
H 3.775223 3.312461 -0.962484
H 3.033947 4.574799 0.017980
C 3.701396 2.838846 1.154539
H 3.636007 1.754898 1.005602
C 2.892358 3.235635 2.417725
H 3.352551 4.134657 2.852139
H 1.879696 3.538855 2.118181
C 2.715310 2.175847 3.542387
C 1.828580 1.015501 3.050184
H 2.271465 0.485086 2.202036
H 0.842812 1.378010 2.737420
H 1.674347 0.281453 3.848426
C 2.009490 2.868719 4.725851
H 2.619015 3.684932 5.130331
H 1.820592 2.158920 5.538580
H 1.044307 3.289962 4.421798
C 4.063362 1.614060 4.032226
H 4.583819 1.056261 3.247287
H 3.905782 0.924485 4.868890
H 4.728164 2.411761 4.382514
C 3.875473 -0.646965 -2.560742
H 4.064543 -0.871525 -3.615867
H 3.393983 0.335850 -2.506810
C 5.226632 -0.593744 -1.796566
H 4.983451 -0.469603 -0.732867
C 6.055397 -1.894468 -1.980171
H 5.413043 -2.666383 -2.414766
H 6.846490 -1.700278 -2.718229
C 6.712458 -2.505681 -0.711486
C 7.648851 -1.502290 -0.011778
H 7.106950 -0.622257 0.351402
H 8.439587 -1.156487 -0.687843
H 8.133973 -1.965693 0.855142
C 7.539955 -3.727817 -1.157879
H 6.907725 -4.471180 -1.655679
H 8.017287 -4.214731 -0.299771
H 8.330656 -3.437089 -1.859263
C 5.630829 -2.980531 0.279829
H 6.088017 -3.488982 1.136509
H 4.935829 -3.676306 -0.200714

H 5.040818 -2.146632 0.675231
C 5.184588 3.228594 1.260354
H 5.296969 4.317836 1.325917
H 5.747589 2.887179 0.385065
H 5.653681 2.797119 2.146939
C 6.010325 0.651100 -2.242926
H 6.966479 0.730632 -1.717441
H 5.444535 1.571701 -2.056463
H 6.226808 0.608024 -3.317103

E = -2079.98374914 Hartree
ZPVE = 575.24666 kcal/mol
Zero-point correction= 0.916714 (Hartree/Particle)
Thermal correction to Energy= 0.967074
Thermal correction to Enthalpy= 0.968018
Thermal correction to Gibbs Free Energy= 0.825098
Sum of electronic and zero-point Energies= -2079.067035
Sum of electronic and thermal Energies= -2079.016675
Sum of electronic and thermal Enthalpies= -2079.015731
Sum of electronic and thermal Free Energies= -2079.158651

Conformer 2 with dispersion
N -7.072097 0.094892 1.165360
N -7.278963 -0.752180 0.266322
C -5.809034 0.757789 1.341062
C -3.453817 2.187816 1.899147
C -5.077322 1.344154 0.298593
C -5.386350 0.933886 2.663742
C -4.204239 1.614635 2.933168
C -3.916549 2.057027 0.583534
H -5.411380 1.239885 -0.726291
H -5.989656 0.516133 3.463157
H -3.863591 1.716710 3.959962
H -3.342654 2.501744 -0.224220
C -6.215256 -1.234426 -0.564139
C -4.156761 -2.068627 -2.273384
C -6.406711 -1.214121 -1.945522
C -5.039601 -1.779885 -0.028971
C -4.028878 -2.195149 -0.881540
C -5.367109 -1.602188 -2.791569
H -7.343414 -0.837017 -2.342745
H -4.912774 -1.834424 1.046284
H -3.096297 -2.566870 -0.466583
H -5.491389 -1.518796 -3.867714
C -2.153468 2.898960 2.192296
H -1.909922 3.570280 1.359877
H -2.257926 3.520488 3.088309
C -2.946150 -2.291431 -3.157555
H -3.236812 -2.192085 -4.209702
H -2.549797 -3.305222 -3.030922
C -0.973723 1.960819 2.370739

C 1.365342 0.409244 2.472901
C -0.811561 0.849804 1.535618
C 0.026022 2.234422 3.310772
C 1.193656 1.473747 3.360757
C 0.339470 0.074002 1.586198
H -1.579468 0.605112 0.810310
H -0.088946 3.078266 3.985103
H 1.982671 1.726900 4.063711
H 0.459537 -0.759477 0.908094
C -1.874747 -1.280137 -2.788480
C -0.046051 0.621739 -1.790435
C -0.619442 -1.673420 -2.319729
C -2.171776 0.088322 -2.797291
C -1.280413 1.038603 -2.313770
C 0.291227 -0.738463 -1.833797
H -0.352867 -2.726935 -2.309505
H -3.143765 0.416773 -3.154448
H -1.533570 2.087683 -2.301513
H 1.253049 -1.057695 -1.449132
N 2.587418 -0.316491 2.473819
H 3.046590 -0.450423 3.362717
N 0.876261 1.499317 -1.193298
H 1.699436 1.036215 -0.825147
C 3.280903 -0.680398 1.355326
C 0.615756 2.749572 -0.690693
O -0.443410 3.350431 -0.862108
O 2.879660 -0.454068 0.211062
C 4.582443 -1.420317 1.590316
H 5.375229 -0.825215 1.123066
H 4.815192 -1.502516 2.659129
C 4.543064 -2.822553 0.946644
H 4.235032 -2.664482 -0.092182
C 3.507527 -3.730298 1.655487
H 4.038542 -4.381240 2.362820
H 2.833602 -3.116441 2.266691
C 2.610838 -4.611224 0.750110
C 1.661842 -3.727796 -0.078013
H 2.207851 -3.045918 -0.734706
H 1.022359 -3.121103 0.571872
H 1.010049 -4.346542 -0.704066
C 1.769789 -5.521686 1.661393
H 2.407813 -6.186824 2.253637
H 1.090505 -6.145931 1.071248
H 1.162385 -4.930934 2.356061
C 3.450836 -5.486712 -0.194075
H 4.036801 -4.883152 -0.893949
H 2.802308 -6.139414 -0.788089
H 4.142040 -6.125323 0.366719
C 1.718788 3.354652 0.167710
H 1.607885 4.439377 0.068478
H 1.452134 3.113017 1.202918

C 3.178315 2.946560 -0.096045
H 3.255366 1.856828 -0.033647
C 3.642793 3.408120 -1.496856
H 2.765114 3.519778 -2.144998
H 4.073530 4.414715 -1.403209
C 4.647592 2.508109 -2.261462
C 5.914947 2.226728 -1.437528
H 5.687766 1.643503 -0.539436
H 6.407822 3.154786 -1.127722
H 6.633283 1.648995 -2.029715
C 5.046195 3.252171 -3.548518
H 4.167408 3.464735 -4.167082
H 5.740868 2.653816 -4.147963
H 5.534313 4.206139 -3.319762
C 3.997367 1.166331 -2.647104
H 4.671827 0.591592 -3.291891
H 3.062547 1.322777 -3.195003
H 3.780881 0.547990 -1.772098
C 5.948138 -3.434662 0.948243
H 6.324378 -3.542351 1.972479
H 6.655452 -2.808343 0.394532
H 5.948566 -4.427016 0.491387
C 4.047142 3.519802 1.033990
H 5.102808 3.273094 0.905538
H 3.723470 3.131236 2.006152
H 3.963274 4.612323 1.068515

E = -2080.23690863 Hartree
ZPVE = 577.28821 kcal/mol
Zero-point correction= 0.919967 (Hartree/Particle)
Thermal correction to Energy= 0.969306
Thermal correction to Enthalpy= 0.970251
Thermal correction to Gibbs Free Energy= 0.834945
Sum of electronic and zero-point Energies= -2079.316941
Sum of electronic and thermal Energies= -2079.267602
Sum of electronic and thermal Enthalpies= -2079.266658
Sum of electronic and thermal Free Energies= -2079.401963

Compound (*S*,*S*)-4 TMH
Conformer 0 without dispersion
H 10.835366 3.445747 -2.226827
C 10.297637 3.205071 -1.302344
H 10.335356 4.088059 -0.654647
H 9.247450 3.028346 -1.561694
H 11.134489 1.050653 -2.549462
H 12.967472 2.466142 -1.191156
H -1.687188 4.764293 -0.214593
C 12.388494 2.281580 -0.278879
C 10.764850 0.761073 -1.555285
C 10.912397 1.978221 -0.598800
H -0.386392 4.521290 1.891117

H 12.718751 -0.818349 -2.906963
H 12.466379 3.176831 0.348249
H 9.686854 0.590928 -1.681052
C -1.663794 3.806237 0.298537
H -3.128494 3.912623 -1.841823
H 13.488130 0.090152 -1.603772
C -0.922010 3.675730 1.470902
C 12.822239 -0.749613 -1.816863
H -5.061829 3.833471 0.304695
H 10.277158 -1.774639 -2.532040
C 11.451776 -0.569944 -1.145387
H 12.865210 1.460710 0.266286
C 10.144039 1.724879 0.713559
H 1.592395 1.640437 1.211450
C -5.491408 3.107463 -0.380081
C -3.213889 2.876960 -1.489042
H 10.248295 2.580149 1.390847
C 10.554395 -1.782381 -1.469440
H 9.075406 1.579571 0.520987
H -7.445448 3.265753 0.492622
C -2.399694 2.731480 -0.214825
H 13.317347 -1.663944 -1.471298
C -6.837527 2.784236 -0.270574
C -4.680613 2.504823 -1.352360
C -0.852268 2.440220 2.123754
H 8.208577 -2.197735 -2.228349
N -0.173745 2.419346 3.387387
C 1.595503 0.646431 1.644666
H 2.618859 -0.236899 -0.018342
H -2.768287 2.255000 -2.275782
H 11.591309 -0.584511 -0.059042
C 9.324904 -1.852162 -0.565715
N 8.142207 -2.104388 -1.225309
H 6.025750 -2.627409 -2.664598
H 10.500523 0.834279 1.238417
H 11.118980 -2.707989 -1.296946
N 0.579326 1.495074 3.764747
C 2.169260 -0.414988 0.954609
C 5.810055 -2.555040 -1.600677
C -7.421053 1.840128 -1.131946
C -2.366187 1.512920 0.481643
C 6.844986 -2.278782 -0.696908
O 9.412821 -1.709097 0.647600
C -5.274470 1.566889 -2.201762
C 1.038531 0.434002 2.916145
C -1.597320 1.357363 1.629081
H -9.237468 2.078578 -0.210285
C 4.505694 -2.737547 -1.148560
N -8.793232 1.562522 -0.955891
H 3.721143 -2.954381 -1.869157
C -6.624871 1.227096 -2.108630

C 6.548188 -2.186331 0.672927
H -4.670148 1.083572 -2.965686
H -11.320608 1.563636 -0.658942
C 4.192986 -2.651893 0.211847
C 2.186225 -1.711341 1.492784
H -2.957419 0.676535 0.118951
H 7.342070 -1.972077 1.373717
C 5.237449 -2.371543 1.103205
H -1.584469 0.407661 2.152255
C -9.603360 0.690950 -1.652256
H -12.702104 0.574058 1.091320
C 1.102061 -0.841217 3.488460
C 2.774999 -2.872852 0.709457
H -7.066300 0.500757 -2.775386
C -11.029597 0.611453 -1.120625
H 2.130639 -3.088563 -0.152634
H -10.470109 -0.410820 0.683978
C 1.642616 -1.904510 2.768471
H 5.022215 -2.289258 2.165362
O -9.213945 0.005631 -2.591672
C -12.610079 -0.385552 0.569817
C -11.232951 -0.539033 -0.095645
H -8.657405 -1.634009 0.764032
H 0.719923 -0.983596 4.494476
H -11.684756 0.452976 -1.982866
H -12.789428 -1.181008 1.298227
H 2.742866 -3.770461 1.339512
H -10.776413 -2.362598 2.057207
H -13.409415 -0.436508 -0.178971
C -8.757055 -2.536987 0.151990
H 1.660915 -2.896112 3.213413
H -8.324822 -2.320429 -0.830354
C -11.054607 -1.933529 -0.755657
H -8.151314 -3.317093 0.626848
C -10.806677 -3.256797 1.425440
C -10.225118 -2.991490 0.023864
H -10.578469 -1.807943 -1.733362
H -10.233772 -4.036914 1.939673
H -12.051624 -2.351791 -0.952528
H -11.847881 -3.594681 1.367837
C -10.264553 -4.302673 -0.786600
H -9.852384 -4.158451 -1.791389
H -9.678846 -5.086917 -0.293819
H -11.291045 -4.671269 -0.895464

E = -2079.97221209 Hartree
ZPVE = 574.62636 kcal/mol
Zero-point correction= 0.915725 (Hartree/Particle)
Thermal correction to Energy= 0.966551
Thermal correction to Enthalpy= 0.967495
Thermal correction to Gibbs Free Energy= 0.817468

Sum of electronic and zero-point Energies= -2079.056487
Sum of electronic and thermal Energies= -2079.005661
Sum of electronic and thermal Enthalpies= -2079.004717
Sum of electronic and thermal Free Energies= -2079.154744

Conformer 0 with dispersion
H 7.740040 3.890518 -1.719941
C 7.311523 3.172796 -1.011348
H 6.814369 3.740662 -0.217674
H 6.544169 2.595043 -1.538855
H 9.347785 2.181310 -2.367235
H 9.932522 3.803256 -0.411816
H -1.126067 4.545382 1.269658
C 9.451608 3.107641 0.285236
C 9.029511 1.461299 -1.602048
C 8.395012 2.248606 -0.429213
H -0.116770 3.615617 3.348746
H 11.534834 1.153839 -2.887048
H 8.990347 3.699502 1.082980
H 8.227149 0.873773 -2.064821
C -1.228907 3.487766 1.496725
H -2.500301 4.315906 -0.617535
H 11.720214 2.117053 -1.416834
C -0.653518 2.974562 2.657105
C 11.548077 1.103821 -1.791720
H -4.679474 3.715293 1.056555
H 9.819472 -0.899990 -2.839240
C 10.228569 0.541108 -1.253157
H 10.232907 2.493734 0.743611
C 7.737143 1.285649 0.574757
H 1.654657 0.961416 2.009303
C -4.990726 3.163034 0.174559
C -2.591404 3.223160 -0.629075
H 7.282745 1.845415 1.399324
C 10.012277 -0.903688 -1.758888
H 6.946617 0.698216 0.095180
H -7.038638 3.052702 0.807589
C -1.958087 2.666485 0.631504
H 12.399510 0.483437 -1.493055
C -6.318627 2.785332 0.037565
C -4.042083 2.825072 -0.799400
C -0.751773 1.610059 2.940352
H 7.744459 -1.474999 -2.646574
N -0.213753 1.168237 4.192930
C 1.548014 -0.113734 2.092537
H 2.597903 -0.517060 0.267792
H -2.024711 2.880187 -1.503679
H 10.315604 0.459613 -0.164663
C 8.892743 -1.568664 -0.974101
N 7.721812 -1.745583 -1.674761
H 5.573023 -1.890236 -3.146913

H 8.452069 0.579056 1.003488
H 10.918772 -1.490949 -1.583826
N 0.442923 0.110685 4.318063
C 2.068919 -0.947627 1.112405
C 5.397022 -2.163189 -2.109068
C -6.739749 2.049481 -1.081590
C -2.101034 1.310152 0.960458
C 6.458324 -2.144451 -1.195683
O 9.032884 -1.857567 0.207978
C -4.475045 2.096127 -1.908253
C 0.891319 -0.667734 3.201736
C -1.499284 0.778374 2.092741
H -8.655968 1.988642 -0.358058
C 4.116856 -2.519494 -1.695178
N -8.097515 1.681362 -1.141106
H 3.305730 -2.523829 -2.418121
C -5.805195 1.706532 -2.066195
C 6.218849 -2.494222 0.142841
H -3.756768 1.820574 -2.675936
H -10.667895 1.337839 -1.224369
C 3.860691 -2.866992 -0.366712
C 1.933768 -2.341188 1.197582
H -2.699373 0.669723 0.319465
H 7.037582 -2.481155 0.846550
C 4.931402 -2.844080 0.535192
H -1.610404 -0.273327 2.329613
C -8.722136 0.832225 -2.027125
H -12.029390 -0.028534 0.348610
C 0.804421 -2.056590 3.328901
C 2.468695 -3.238335 0.097645
H -6.126473 1.140816 -2.927783
C -10.137878 0.461236 -1.618035
H 1.786372 -3.204841 -0.761322
H -9.568528 -0.300137 0.302134
C 1.297294 -2.880475 2.319896
H 4.751511 -3.096042 1.576525
O -8.176279 0.359607 -3.017267
C -11.573714 -0.930042 -0.075692
C -10.142528 -0.668292 -0.557776
H -7.424132 -0.644717 0.400374
H 0.342562 -2.472671 4.218232
H -10.659487 0.130064 -2.519551
H -11.588126 -1.709815 0.691396
H 2.456979 -4.275899 0.452413
H -9.191516 -2.001653 1.720413
H -12.205626 -1.267530 -0.904994
C -7.151383 -1.564603 -0.125404
H 1.204510 -3.958875 2.414934
H -6.791022 -1.281617 -1.118927
C -9.467502 -1.957546 -1.092814
H -6.318791 -2.016682 0.424234

C -8.841132 -2.891250 1.186709
C -8.330833 -2.548879 -0.222861
H -9.057240 -1.768878 -2.089904
H -8.043972 -3.340387 1.788959
H -10.236895 -2.729935 -1.219961
H -9.670547 -3.606061 1.142802
C -7.840871 -3.837947 -0.904725
H -7.463679 -3.629272 -1.911544
H -7.030715 -4.301160 -0.331100
H -8.650794 -4.570537 -0.993487

E = -2080.20438023 Hartree
ZPVE = 576.16900 kcal/mol
Zero-point correction= 0.918184 (Hartree/Particle)
Thermal correction to Energy= 0.968501
Thermal correction to Enthalpy= 0.969446
Thermal correction to Gibbs Free Energy= 0.821861
Sum of electronic and zero-point Energies= -2079.286196
Sum of electronic and thermal Energies= -2079.235879
Sum of electronic and thermal Enthalpies= -2079.234935
Sum of electronic and thermal Free Energies= -2079.382519

Conformer 1 without dispersion
N 7.515599 0.346920 -0.337690
N 7.444536 0.387318 0.910024
C 6.384617 0.126353 -1.193859
C 4.398024 -0.405489 -3.122303
C 5.508468 -0.956614 -1.027037
C 6.288514 0.915548 -2.345416
C 5.283801 0.668982 -3.277636
C 4.537948 -1.219064 -1.988459
H 5.599204 -1.594854 -0.155012
H 6.997443 1.724976 -2.489572
H 5.201154 1.308171 -4.153147
H 3.869490 -2.064219 -1.851866
C 6.206452 0.438548 1.631951
C 3.936425 0.626781 3.292189
C 6.106812 -0.350288 2.783200
C 5.190625 1.361317 1.338500
C 4.079343 1.455150 2.168378
C 4.966752 -0.277563 3.580515
H 6.919409 -1.026950 3.029100
H 5.281089 2.008016 0.472747
H 3.297735 2.172010 1.934561
H 4.884282 -0.920844 4.452979
C 3.366392 -0.712285 -4.196324
H 3.253984 0.175780 -4.831621
H 3.759997 -1.497023 -4.856103
C 2.720513 0.737931 4.197464
H 2.838775 1.612542 4.850814
H 2.707322 -0.131401 4.868025

C 1.999647 -1.145015 -3.697584
C -0.579868 -1.911455 -2.831318
C 1.278200 -0.369952 -2.779622
C 1.400332 -2.318205 -4.168868
C 0.129931 -2.699412 -3.746302
C 0.006146 -0.733336 -2.343968
H 1.720731 0.541896 -2.388687
H 1.931434 -2.945421 -4.879981
H -0.311010 -3.618064 -4.127120
H -0.530845 -0.118023 -1.637417
C 1.389898 0.851066 3.474738
C -1.106432 1.050820 2.152784
C 1.022697 -0.070050 2.482971
C 0.479253 1.865541 3.784303
C -0.757103 1.977089 3.146131
C -0.199388 0.024929 1.830121
H 1.709373 -0.866367 2.208381
H 0.736441 2.595793 4.548221
H -1.448087 2.767647 3.400439
H -0.460306 -0.690849 1.056215
N -1.875228 -2.353463 -2.466445
H -2.216653 -3.141833 -2.997121
N -2.320912 1.082543 1.439271
H -2.425032 0.360838 0.732080
C -2.718395 -1.883369 -1.498336
C -3.357181 1.978350 1.551554
O -3.384491 2.906191 2.357165
O -2.430253 -0.952868 -0.743045
C -4.070681 -2.579269 -1.418304
H -4.820012 -1.816672 -1.664235
H -4.162743 -3.369559 -2.174366
C -4.374519 -3.156824 -0.015830
H -4.155510 -2.357007 0.700737
C -3.471526 -4.381458 0.288713
H -4.006777 -5.289707 -0.023271
H -2.574950 -4.336100 -0.344818
C -2.966447 -4.578475 1.746552
C -4.122272 -4.610264 2.764310
H -3.737963 -4.818475 3.768940
H -4.851117 -5.391755 2.521058
H -4.651316 -3.653225 2.812522
C -1.981509 -3.456408 2.128632
H -1.598079 -3.607807 3.143415
H -2.447498 -2.466972 2.101689
H -1.122374 -3.437563 1.448659
C -2.222303 -5.928279 1.801419
H -1.391159 -5.953412 1.087246
H -1.807562 -6.105955 2.799631
H -2.892817 -6.762487 1.565056
C -4.476759 1.752526 0.540780
H -4.526824 0.693725 0.263526

H -5.416581 2.019802 1.034775
C -4.306720 2.602833 -0.747884
C -4.422909 4.123741 -0.456280
H -5.428079 4.457498 -0.750623
H -4.351993 4.284896 0.624130
C -3.381087 5.060998 -1.127642
C -3.390577 4.922296 -2.661868
H -3.106448 3.914314 -2.982522
H -2.679661 5.621241 -3.117672
H -4.381744 5.141648 -3.075834
C -1.966127 4.763435 -0.591487
H -1.937015 4.832931 0.500666
H -1.625110 3.759813 -0.867223
H -1.240265 5.476435 -0.999219
C -3.751518 6.511403 -0.758100
H -3.037949 7.222226 -1.190353
H -3.749938 6.654176 0.328089
H -4.749468 6.772903 -1.128730
C -5.871780 -3.493409 0.072294
H -6.149926 -4.234279 -0.687343
H -6.488485 -2.602647 -0.089510
H -6.136053 -3.908452 1.047022
H -3.304849 2.384109 -1.139464
C -5.325923 2.137360 -1.800069
H -5.238178 2.715607 -2.724298
H -5.185457 1.079567 -2.051536
H -6.350664 2.260476 -1.429177

E = -2079.98392745 Hartree
ZPVE = 575.23932 kcal/mol
Zero-point correction= 0.916702 (Hartree/Particle)
Thermal correction to Energy= 0.967110
Thermal correction to Enthalpy= 0.968054
Thermal correction to Gibbs Free Energy= 0.824252
Sum of electronic and zero-point Energies= -2079.067225
Sum of electronic and thermal Energies= -2079.016818
Sum of electronic and thermal Enthalpies= -2079.015874
Sum of electronic and thermal Free Energies= -2079.159676

Conformer 1 with dispersion
N 7.150308 -0.175638 -1.303328
N 7.263613 -1.336945 -0.850793
C 5.926769 0.571100 -1.223040
C 3.692974 2.274083 -1.134460
C 5.539028 1.283793 -2.362759
C 5.218118 0.738360 -0.026417
C 4.119615 1.591173 0.011363
C 4.413443 2.099650 -2.322636
H 6.124165 1.178590 -3.270545
H 5.530465 0.208978 0.865865
H 3.580300 1.727495 0.944443

H 4.101251 2.629901 -3.218165
C 6.132519 -2.083917 -0.387236
C 4.011673 -3.652192 0.574104
C 4.967027 -2.234590 -1.152969
C 6.263206 -2.773741 0.819088
C 5.195057 -3.526333 1.306391
C 3.927046 -3.015449 -0.673263
H 4.880671 -1.731086 -2.108729
H 7.188730 -2.684539 1.378521
H 5.284853 -4.025328 2.267257
H 3.016373 -3.108770 -1.256955
C 2.488957 3.183950 -1.089065
H 2.353944 3.547019 -0.063573
H 2.676158 4.072171 -1.702414
C 2.816076 -4.413086 1.117949
H 3.070872 -4.838297 2.094250
H 2.577948 -5.255059 0.457226
C 1.183003 2.552598 -1.539386
C -1.335112 1.482787 -2.221370
C 0.888470 1.202733 -1.321467
C 0.199027 3.350343 -2.135078
C -1.046095 2.830421 -2.465594
C -0.349740 0.660394 -1.662722
H 1.636335 0.553316 -0.878257
H 0.403938 4.399756 -2.326377
H -1.802905 3.474934 -2.905285
H -0.544576 -0.387075 -1.492018
C 1.611705 -3.502120 1.231191
C -0.484261 -1.616701 1.316199
C 0.613264 -3.498012 0.253314
C 1.522283 -2.562922 2.264831
C 0.490803 -1.631491 2.325836
C -0.423407 -2.570832 0.288591
H 0.647656 -4.226984 -0.552339
H 2.289312 -2.548171 3.034923
H 0.424114 -0.918318 3.134331
H -1.191306 -2.574897 -0.478332
N -2.625452 1.023790 -2.582657
H -3.145215 1.644359 -3.185942
N -1.511688 -0.663058 1.243935
H -2.009200 -0.644808 0.357776
C -3.292982 -0.081840 -2.141811
C -1.885833 0.263730 2.179758
O -1.417761 0.330071 3.314509
O -2.811667 -0.880474 -1.334072
C -4.683386 -0.262456 -2.727075
H -5.005627 0.665610 -3.213595
H -4.617154 -1.019650 -3.519901
C -5.741233 -0.705110 -1.689523
H -5.587559 -0.099811 -0.787636
C -5.587543 -2.202268 -1.327972

H -6.230391 -2.782236 -2.005528
H -4.559369 -2.510824 -1.530852
C -5.900355 -2.624141 0.130595
C -7.317832 -2.215154 0.563261
H -7.532793 -2.594161 1.568203
H -8.076609 -2.621162 -0.114904
H -7.434811 -1.127324 0.594976
C -4.877922 -2.011107 1.102748
H -5.068478 -2.353169 2.125503
H -4.929193 -0.918463 1.111540
H -3.857031 -2.293556 0.835344
C -5.783796 -4.157405 0.199586
H -4.783410 -4.488551 -0.099332
H -5.965160 -4.517575 1.217773
H -6.511208 -4.640222 -0.462634
C -2.893990 1.274459 1.653608
H -3.423500 0.867871 0.786283
H -3.631379 1.464978 2.439783
C -2.196380 2.600342 1.258627
C -1.662867 3.347487 2.506912
H -2.329605 4.193786 2.722087
H -1.716834 2.679220 3.370228
C -0.211216 3.878288 2.432384
C -0.040797 4.861799 1.263693
H -0.234494 4.380745 0.301609
H 0.979897 5.261074 1.237177
H -0.725544 5.711934 1.362607
C 0.778388 2.707421 2.283938
H 0.643902 1.980854 3.089526
H 0.644222 2.175614 1.337626
H 1.811009 3.075026 2.317868
C 0.090048 4.611165 3.750843
H 1.112710 5.004686 3.756476
H -0.015744 3.936463 4.606928
H -0.594374 5.454014 3.899442
C -7.134289 -0.386585 -2.251368
H -7.290412 -0.905205 -3.204720
H -7.257995 0.687225 -2.430221
H -7.924859 -0.705917 -1.570657
H -1.356835 2.334289 0.606766
C -3.157870 3.464457 0.434662
H -2.682591 4.401342 0.130738
H -3.482995 2.940983 -0.471343
H -4.051274 3.716593 1.018033

E = -2080.23655379 Hartree
ZPVE = 577.49143 kcal/mol
Zero-point correction= 0.920291 (Hartree/Particle)
Thermal correction to Energy= 0.969569
Thermal correction to Enthalpy= 0.970513
Thermal correction to Gibbs Free Energy= 0.835544

Sum of electronic and zero-point Energies= -2079.316263
Sum of electronic and thermal Energies= -2079.266985
Sum of electronic and thermal Enthalpies= -2079.266041
Sum of electronic and thermal Free Energies= -2079.401010

Conformer 2 without dispersion
N 7.515614 0.346961 -0.337685
N 7.444545 0.387372 0.910028
C 6.384635 0.126393 -1.193857
C 4.398043 -0.405454 -3.122301
C 5.508480 -0.956568 -1.027029
C 6.288538 0.915580 -2.345421
C 5.283826 0.669012 -3.277641
C 4.537961 -1.219021 -1.988451
H 5.599211 -1.594802 -0.154998
H 6.997471 1.725003 -2.489581
H 5.201184 1.308195 -4.153156
H 3.869499 -2.064172 -1.851853
C 6.206459 0.438619 1.631949
C 3.936426 0.626881 3.292176
C 6.106810 -0.350204 2.783206
C 5.190638 1.361390 1.338484
C 4.079353 1.455237 2.168357
C 4.966747 -0.277465 3.580516
H 6.919403 -1.026867 3.029117
H 5.281108 2.008079 0.472725
H 3.297749 2.172099 1.934529
H 4.884270 -0.920736 4.452986
C 3.366411 -0.712251 -4.196322
H 3.253991 0.175820 -4.831610
H 3.760023 -1.496977 -4.856111
C 2.720511 0.738046 4.197445
H 2.838763 1.612679 4.850768
H 2.707327 -0.131265 4.868032
C 1.999673 -1.145001 -3.697582
C -0.579827 -1.911484 -2.831310
C 1.278208 -0.369942 -2.779631
C 1.400382 -2.318209 -4.168853
C 0.129989 -2.699437 -3.746283
C 0.006161 -0.733346 -2.343973
H 1.720721 0.541920 -2.388707
H 1.931497 -2.945422 -4.879959
H -0.310934 -3.618103 -4.127091
H -0.530842 -0.118035 -1.637431
C 1.389896 0.851147 3.474713
C -1.106439 1.050833 2.152758
C 1.022715 -0.069985 2.482954
C 0.479228 1.865604 3.784269
C -0.757131 1.977119 3.146096
C -0.199372 0.024960 1.830103
H 1.709409 -0.866289 2.208371

H 0.736399 2.595869 4.548180
H -1.448133 2.767663 3.400398
H -0.460274 -0.690831 1.056204
N -1.875178 -2.353514 -2.466431
H -2.216587 -3.141897 -2.997098
N -2.320921 1.082522 1.439246
H -2.425024 0.360809 0.732060
C -2.718354 -1.883427 -1.498327
C -3.357214 1.978299 1.551528
O -3.384549 2.906142 2.357137
O -2.430230 -0.952910 -0.743046
C -4.070625 -2.579353 -1.418284
H -4.819972 -1.816775 -1.664224
H -4.162672 -3.369654 -2.174337
C -4.374450 -3.156897 -0.015803
H -4.155458 -2.357066 0.700753
C -3.471429 -4.381507 0.288755
H -4.006660 -5.289772 -0.023220
H -2.574853 -4.336136 -0.344774
C -2.966350 -4.578498 1.746598
C -2.222175 -5.928285 1.801480
H -1.807432 -6.105941 2.799694
H -1.391030 -5.953407 1.087309
H -2.892671 -6.762510 1.565124
C -4.122176 -4.610302 2.764354
H -4.851003 -5.391811 2.521108
H -4.651240 -3.653274 2.812556
H -3.737864 -4.818496 3.768986
C -1.981437 -3.456405 2.128669
H -2.447448 -2.466980 2.101714
H -1.598007 -3.607785 3.143454
H -1.122300 -3.437548 1.448698
C -4.476789 1.752441 0.540758
H -4.526818 0.693640 0.263498
H -5.416618 2.019682 1.034758
C -4.306785 2.602762 -0.747902
C -4.423035 4.123663 -0.456290
H -5.428220 4.457380 -0.750626
H -4.352119 4.284816 0.624120
C -3.381256 5.060967 -1.127654
C -3.390747 4.922272 -2.661880
H -3.106577 3.914305 -2.982541
H -2.679863 5.621250 -3.117684
H -4.381926 5.141584 -3.075840
C -1.966281 4.763460 -0.591507
H -1.937166 4.832950 0.500646
H -1.625223 3.759854 -0.867252
H -1.240450 5.476493 -0.999238
C -3.751745 6.511355 -0.758102
H -3.038207 7.222209 -1.190355
H -3.750165 6.654123 0.328087

H -4.749708 6.772815 -1.128726
C -5.871703 -3.493514 0.072325
H -6.149833 -4.234399 -0.687303
H -6.488428 -2.602767 -0.089490
H -6.135967 -3.908550 1.047059
H -3.304905 2.384080 -1.139483
C -5.325969 2.137252 -1.800089
H -5.185460 1.079466 -2.051561
H -6.350715 2.260324 -1.429195
H -5.238248 2.715508 -2.724315

E = -2079.98392745 Hartree
ZPVE = 575.23936 kcal/mol
Zero-point correction= 0.916702 (Hartree/Particle)
Thermal correction to Energy= 0.967110
Thermal correction to Enthalpy= 0.968054
Thermal correction to Gibbs Free Energy= 0.824252
Sum of electronic and zero-point Energies= -2079.067225
Sum of electronic and thermal Energies= -2079.016818
Sum of electronic and thermal Enthalpies= -2079.015874
Sum of electronic and thermal Free Energies= -2079.159676

Conformer 2 with dispersion
N 6.065291 -1.503962 -2.103216
N 6.276940 -2.268464 -1.134950
C 4.960306 -0.587953 -2.131306
C 2.891496 1.294069 -2.387096
C 4.218740 -0.515156 -3.315305
C 4.700961 0.314693 -1.092947
C 3.680826 1.250641 -1.231781
C 3.173989 0.395931 -3.424043
H 4.462254 -1.190777 -4.128813
H 5.292874 0.279948 -0.185993
H 3.486129 1.953522 -0.426558
H 2.576623 0.424366 -4.331224
C 5.335095 -2.414368 -0.064377
C 3.597291 -2.768545 2.112019
C 3.974932 -2.676642 -0.286527
C 5.834443 -2.402685 1.238822
C 4.961892 -2.548689 2.316794
C 3.124576 -2.854919 0.793702
H 3.592669 -2.723829 -1.299395
H 6.896607 -2.243388 1.393644
H 5.348236 -2.493847 3.330749
H 2.067107 -3.029442 0.619141
C 1.747599 2.273340 -2.507687
H 1.910481 3.104968 -1.814291
H 1.735559 2.709657 -3.512238
C 2.625882 -2.873458 3.273316
H 3.170466 -2.730819 4.212788
H 2.194917 -3.881241 3.308909

C 0.384786 1.666070 -2.226033
C -2.172609 0.621399 -1.678158
C 0.181385 0.772419 -1.167019
C -0.719208 2.021047 -3.008060
C -1.986182 1.514570 -2.737006
C -1.077546 0.245186 -0.891684
H 1.020362 0.465899 -0.550526
H -0.588445 2.710816 -3.836769
H -2.833438 1.811426 -3.349346
H -1.201325 -0.456514 -0.080736
C 1.513412 -1.854872 3.141889
C -0.474106 0.091447 2.673188
C 0.234741 -2.227104 2.719138
C 1.766827 -0.493421 3.349098
C 0.796284 0.478546 3.128918
C -0.750346 -1.271737 2.490018
H 0.003513 -3.277536 2.561672
H 2.756435 -0.183377 3.674909
H 0.998959 1.525666 3.301204
H -1.741128 -1.568772 2.164603
N -3.468397 0.076805 -1.494841
H -4.054245 0.064409 -2.316664
N -1.486945 0.999153 2.328745
H -2.237118 0.602349 1.768507
C -4.022504 -0.428458 -0.355172
C -1.540863 2.347343 2.562878
O -0.732087 2.961902 3.255218
O -3.471328 -0.398597 0.747002
C -5.365028 -1.111282 -0.525369
H -6.034618 -0.698235 0.235335
H -5.807397 -0.908910 -1.508509
C -5.223281 -2.637142 -0.312596
H -4.699332 -2.754565 0.641879
C -4.388205 -3.296621 -1.439793
H -5.069918 -3.826130 -2.118103
H -3.904573 -2.523552 -2.049503
C -3.275109 -4.279012 -0.997283
C -2.666505 -4.906317 -2.263100
H -1.851721 -5.591687 -2.006859
H -2.258520 -4.136135 -2.927117
H -3.417484 -5.472729 -2.824793
C -3.843066 -5.396785 -0.107359
H -4.634780 -5.950385 -0.624067
H -4.257889 -5.003042 0.825693
H -3.056863 -6.110871 0.159580
C -2.170619 -3.523922 -0.238129
H -2.554980 -3.037684 0.661553
H -1.373491 -4.209653 0.068325
H -1.720251 -2.748265 -0.866585
C -2.665509 3.038617 1.806818
H -3.422191 2.309944 1.498301

H -3.144775 3.759799 2.476472
C -2.108522 3.774422 0.561091
C -1.305919 5.034557 0.972509
H -1.928366 5.921189 0.788380
H -1.119891 5.000045 2.049089
C 0.063393 5.247299 0.284151
C -0.085345 5.311982 -1.244400
H -0.485664 4.379359 -1.650189
H 0.884606 5.494061 -1.721710
H -0.757106 6.126176 -1.539547
C 1.033379 4.116091 0.672892
H 1.128600 4.043138 1.759173
H 0.689770 3.142673 0.311680
H 2.027344 4.302325 0.248536
C 0.641185 6.581652 0.786255
H 1.622452 6.777723 0.339618
H 0.763061 6.570873 1.874570
H -0.017590 7.418796 0.529185
C -6.608880 -3.277846 -0.178442
H -7.198718 -3.126300 -1.090122
H -7.167881 -2.849389 0.659861
H -6.527020 -4.355405 -0.013769
H -1.444047 3.070625 0.046886
C -3.253555 4.106106 -0.402797
H -3.765415 3.196710 -0.735911
H -3.993757 4.754486 0.080885
H -2.883067 4.627425 -1.289897

E = -2080.23827493 Hartree
ZPVE = 577.52303 kcal/mol
Zero-point correction= 0.920342 (Hartree/Particle)
Thermal correction to Energy= 0.969614
Thermal correction to Enthalpy= 0.970559
Thermal correction to Gibbs Free Energy= 0.835458
Sum of electronic and zero-point Energies= -2079.317933
Sum of electronic and thermal Energies= -2079.268661
Sum of electronic and thermal Enthalpies= -2079.267716
Sum of electronic and thermal Free Energies= -2079.402817

Compound 5 CO^{t}Bu
Conformer 0 without dispersion
H -2.421517 1.713033 3.804064
H -3.679610 -0.236475 3.552083
C -2.201785 1.735882 2.739707
C -3.513347 -0.425885 2.484292
H -2.919976 -1.348079 2.432584
H -1.103718 3.595861 2.854457
C -1.452106 2.788170 2.218343
C -4.846087 -0.668030 1.797519
C -2.698847 0.722358 1.911431
H -5.613137 1.307543 2.176055

C -5.823544 0.334498 1.739868
C -5.147976 -1.902934 1.214633
H -4.414428 -2.704879 1.240919
C -7.060099 0.129536 1.134254
C -6.378052 -2.128722 0.602428
H -7.803325 0.912703 1.100635
C -7.347884 -1.117630 0.555567
C -1.130922 2.814335 0.856741
H 1.432793 1.880861 1.031353
H -11.946593 -1.816309 0.821790
C -2.418697 0.792510 0.537827
H -6.586511 -3.099248 0.156932
H 8.657159 -2.295285 0.521488
N -8.570537 -1.421919 -0.081920
O -9.745206 0.530293 0.147557
N -0.462365 3.989355 0.377415
C 9.706489 -0.573700 0.231772
N 8.593330 -1.377951 0.105947
H 2.820365 0.035353 0.159422
C -1.637388 1.814365 0.010974
H 6.611083 -3.071125 -0.033592
C -12.089560 -1.175927 -0.055646
C -9.677042 -0.624695 -0.256849
H -10.505792 -3.387357 -0.609168
C 1.635324 1.810902 -0.031614
H -2.825880 0.036293 -0.127593
H -12.231522 -0.150391 0.291050
H -8.627431 -2.356901 -0.454547
C 6.394524 -2.121565 -0.518668
O 9.768296 0.559148 -0.226560
C 7.362522 -1.107484 -0.528237
C 2.413150 0.771445 -0.528222
C -10.878221 -1.254464 -1.010051
H -12.998633 -1.504870 -0.570112
N 0.464017 3.975767 -0.462442
C -10.670299 -2.712476 -1.457683
C 5.156388 -1.925575 -1.124986
H 4.424998 -2.729742 -1.106850
C 1.129232 2.785299 -0.906973
C 7.063606 0.112294 -1.158094
H -1.432540 1.850755 -1.053204
H -11.566342 -3.068412 -1.975536
H 7.804528 0.898278 -1.168966
C -11.137847 -0.367264 -2.247169
H -9.836074 -2.815410 -2.161979
C 4.843651 -0.718413 -1.757934
H -11.262095 0.674766 -1.945654
C 5.819199 0.287635 -1.756596
C 2.689743 0.657824 -1.899666
H 2.913008 -1.430668 -2.342299
H -12.044792 -0.696360 -2.765331

C 3.501675 -0.508975 -2.437572
C 1.447194 2.716185 -2.267776
H -10.304566 -0.420365 -2.956557
H 5.601046 1.240163 -2.232516
C 2.192998 1.646205 -2.757909
H 1.099007 3.504415 -2.927962
H 3.654042 -0.360744 -3.513893
H 2.409807 1.589495 -3.821598
C 10.885736 -1.218560 1.006517
C 11.359778 -2.498085 0.280511
H 12.234907 -2.912997 0.791663
H 10.595138 -3.282444 0.260092
H 11.644918 -2.282460 -0.753924
C 12.037874 -0.202245 1.052136
H 11.730068 0.719288 1.551469
H 12.887561 -0.628762 1.595192
H 12.365405 0.066862 0.045294
C 10.445892 -1.559659 2.448461
H 11.297611 -1.952000 3.014088
H 10.076651 -0.670626 2.969131
H 9.656930 -2.318982 2.482518

E = -1765.44783138 Hartree
ZPVE = 431.67631 kcal/mol
Zero-point correction= 0.687920 (Hartree/Particle)
Thermal correction to Energy= 0.728463
Thermal correction to Enthalpy= 0.729408
Thermal correction to Gibbs Free Energy= 0.603592
Sum of electronic and zero-point Energies= -1764.759911
Sum of electronic and thermal Energies= -1764.719368
Sum of electronic and thermal Enthalpies= -1764.718424
Sum of electronic and thermal Free Energies= -1764.844239

Conformer 0 with dispersion
H -2.184810 1.736493 3.861805
H -3.427023 -0.232557 3.613382
C -2.015990 1.789328 2.789765
C -3.264826 -0.406671 2.542847
H -2.639004 -1.305366 2.473248
H -0.975164 3.684072 2.887452
C -1.330570 2.877664 2.254413
C -4.588875 -0.677816 1.859164
C -2.512709 0.775797 1.964760
H -5.368757 1.293837 2.213550
C -5.571569 0.316670 1.784286
C -4.867957 -1.917416 1.278985
H -4.123854 -2.708438 1.316124
C -6.797772 0.097388 1.165645
C -6.087777 -2.157580 0.653402
H -7.548483 0.871738 1.115643
C -7.064426 -1.155470 0.591070

C -1.080089 2.938082 0.881365
H 1.435588 2.035734 1.007244
H -11.647378 -1.879040 0.799673
C -2.307883 0.880959 0.580978
H -6.283356 -3.130194 0.207642
H 8.351025 -2.343835 0.502776
N -8.273875 -1.472292 -0.059728
O -9.461919 0.473946 0.143623
N -0.449234 4.127484 0.390619
C 9.413094 -0.630937 0.224608
N 8.297901 -1.426476 0.086928
H 2.719835 0.122476 0.109065
C -1.591954 1.939379 0.038876
H 6.306621 -3.100034 -0.071938
C -11.785017 -1.248317 -0.085083
C -9.379940 -0.682761 -0.252939
H -10.175754 -3.446169 -0.589832
C 1.589715 1.936355 -0.060834
H -2.724833 0.122953 -0.075310
H -11.939055 -0.220615 0.248576
H -8.320394 -2.408937 -0.428209
C 6.103130 -2.149973 -0.560722
O 9.490673 0.501613 -0.231545
C 7.079280 -1.145271 -0.559997
C 2.301904 0.859396 -0.570272
C -10.560606 -1.325281 -1.017944
H -12.682192 -1.590347 -0.610896
N 0.449184 4.113263 -0.479786
C -10.332972 -2.782666 -1.448135
C 4.874291 -1.941159 -1.180006
H 4.131313 -2.734062 -1.168949
C 1.077058 2.907926 -0.934026
C 6.801881 0.078420 -1.190359
H -1.434562 2.003348 -1.031398
H -11.217419 -3.150277 -1.976619
H 7.551466 0.855483 -1.188825
C -10.804031 -0.451664 -2.264408
H -9.486319 -2.882085 -2.137362
C 4.584752 -0.731220 -1.814896
H -10.942528 0.590661 -1.971587
C 5.566839 0.266607 -1.801306
C 2.501594 0.708340 -1.950572
H 2.629753 -1.391139 -2.376897
H -11.697555 -0.793656 -2.796482
C 3.250550 -0.493599 -2.491347
C 1.323017 2.802285 -2.305092
H -9.955479 -0.503897 -2.954901
H 5.356608 1.222025 -2.273857
C 2.004241 1.695324 -2.806720
H 0.966903 3.588124 -2.963113
H 3.397498 -0.361929 -3.570082

H 2.169043 1.606764 -3.877024
C 10.570865 -1.288297 1.011344
C 11.031461 -2.573449 0.292784
H 11.895681 -2.999411 0.812380
H 10.254482 -3.344883 0.267897
H 11.326756 -2.361741 -0.739146
C 11.734912 -0.290774 1.070209
H 11.432061 0.635083 1.563680
H 12.570317 -0.729379 1.624812
H 12.077445 -0.029357 0.066715
C 10.105489 -1.620294 2.444341
H 10.943107 -2.023293 3.022549
H 9.740437 -0.724491 2.955364
H 9.305440 -2.367787 2.464899

E = -1765.62915720 Hartree
ZPVE = 432.51927 kcal/mol
Zero-point correction= 0.689263 (Hartree/Particle)
Thermal correction to Energy= 0.729663
Thermal correction to Enthalpy= 0.730607
Thermal correction to Gibbs Free Energy= 0.605108
Sum of electronic and zero-point Energies= -1764.939894
Sum of electronic and thermal Energies= -1764.899494
Sum of electronic and thermal Enthalpies= -1764.898550
Sum of electronic and thermal Free Energies= -1765.024049

Conformer 1 without dispersion
N -6.578308 1.192966 -0.065011
N -6.718454 0.084379 0.498181
C -5.325441 1.642201 -0.605613
C -2.996261 2.701644 -1.782366
C -4.616091 0.897223 -1.555596
C -4.906791 2.942307 -0.295298
C -3.736268 3.447318 -0.853257
C -3.473557 1.434962 -2.143182
H -4.961948 -0.092014 -1.834943
H -5.495319 3.534081 0.399058
H -3.396936 4.441398 -0.573384
H -2.938916 0.853957 -2.890376
C -5.613951 -0.764552 0.844232
C -3.611985 -2.571651 1.663291
C -5.772981 -2.137768 0.632387
C -4.477909 -0.300258 1.526072
C -3.500546 -1.198830 1.932359
C -4.765546 -3.024188 1.012116
H -6.679536 -2.494154 0.153072
H -4.368131 0.756633 1.740040
H -2.621671 -0.826266 2.450864
H -4.885688 -4.085678 0.811527
C -1.702155 3.242404 -2.361677
H -1.512478 2.753796 -3.325727

H -1.810815 4.311424 -2.575670
C -2.498750 -3.527514 2.067907
H -2.814011 -4.554106 1.851713
H -2.348255 -3.469554 3.153072
C -0.491544 3.044543 -1.457408
C 1.785667 2.661535 0.173511
C -0.153031 1.778343 -0.962857
C 0.332023 4.119758 -1.107535
C 1.456430 3.935641 -0.306203
C 0.964395 1.574625 -0.157927
H -0.781927 0.924437 -1.196425
H 0.098810 5.117614 -1.469896
H 2.083768 4.788101 -0.054715
H 1.193397 0.587230 0.213475
C -1.184688 -3.229758 1.365760
C 1.198278 -2.558915 -0.012086
C -0.183290 -2.472473 1.983956
C -0.954202 -3.659855 0.053471
C 0.213473 -3.343236 -0.636601
C 0.989235 -2.137926 1.311828
H -0.316188 -2.139400 3.010726
H -1.710981 -4.255423 -0.451793
H 0.377121 -3.692964 -1.645320
H 1.752250 -1.545648 1.808227
N 2.925259 2.552556 1.009080
H 3.281357 3.435213 1.345191
N 2.392426 -2.150540 -0.641918
H 2.905630 -1.433388 -0.142064
C 3.644435 1.443789 1.363720
C 2.909873 -2.582363 -1.842929
O 2.359548 -3.420890 -2.548115
O 3.365114 0.309550 0.972216
C 4.830410 1.713924 2.324950
C 5.598166 0.399526 2.537568
H 4.944864 -0.380548 2.933983
C 4.246196 -1.915381 -2.261418
C 3.983284 -0.426983 -2.587944
H 3.627019 0.128565 -1.716212
H 6.023213 0.031062 1.600413
H 6.416850 0.562048 3.245605
H 3.237614 -0.327523 -3.383431
H 4.908890 0.046321 -2.934785
C 4.764216 -2.632732 -3.518169
H 5.694739 -2.163729 -3.855374
H 4.032674 -2.585677 -4.327453
H 4.960849 -3.689374 -3.317664
C 5.297284 -2.031031 -1.135111
H 6.255085 -1.629107 -1.484182
H 5.458275 -3.075763 -0.849583
H 5.015281 -1.470957 -0.238934
C 4.275688 2.209102 3.681343

H 3.741194 3.160560 3.592470
H 5.099281 2.357565 4.387297
H 3.587269 1.477588 4.115770
C 5.787659 2.770258 1.727252
H 6.145893 2.468813 0.738086
H 6.659156 2.887389 2.379175
H 5.328220 3.760529 1.631744

E = -1765.45715757 Hartree
ZPVE = 432.25965 kcal/mol
Zero-point correction= 0.688850 (Hartree/Particle)
Thermal correction to Energy= 0.728944
Thermal correction to Enthalpy= 0.729888
Thermal correction to Gibbs Free Energy= 0.611852
Sum of electronic and zero-point Energies= -1764.768308
Sum of electronic and thermal Energies= -1764.728214
Sum of electronic and thermal Enthalpies= -1764.727270
Sum of electronic and thermal Free Energies= -1764.845305

Conformer 1 with dispersion
N -6.063399 1.115628 1.527422
N -6.467774 -0.030399 1.223136
C -4.735747 1.573110 1.219512
C -2.185171 2.651966 0.740283
C -4.149061 1.456510 -0.047801
C -4.070086 2.281628 2.225906
C -2.795013 2.786128 1.993963
C -2.888966 1.999230 -0.279065
H -4.670867 0.936587 -0.841808
H -4.557684 2.405680 3.187416
H -2.265967 3.299822 2.792066
H -2.427379 1.896476 -1.256482
C -5.573491 -1.008927 0.675852
C -3.832320 -2.844931 -0.528501
C -5.948936 -1.652780 -0.502630
C -4.380501 -1.367467 1.319398
C -3.527705 -2.280286 0.719529
C -5.064437 -2.543236 -1.113149
H -6.899221 -1.401282 -0.962182
H -4.112778 -0.895902 2.258013
H -2.578024 -2.516879 1.190856
H -5.326436 -2.989346 -2.068577
C -0.783586 3.162933 0.504393
H -0.607230 3.263427 -0.573215
H -0.667253 4.159740 0.944109
C -2.773151 -3.637891 -1.266522
H -3.194132 -4.031963 -2.198889
H -2.449348 -4.501960 -0.675198
C 0.297828 2.249350 1.051083
C 2.433023 0.576412 1.775124
C 0.188860 0.858840 0.940473

C 1.470203 2.781815 1.598312
C 2.537710 1.957732 1.950246
C 1.240436 0.025984 1.304406
H -0.716189 0.421013 0.534934
H 1.565538 3.857589 1.716000
H 3.461965 2.387530 2.326402
H 1.152111 -1.044975 1.180894
C -1.577897 -2.742862 -1.545941
C 0.556933 -0.913782 -1.836916
C -0.296134 -3.058039 -1.090026
C -1.755896 -1.513490 -2.191302
C -0.715592 -0.605003 -2.345203
C 0.763175 -2.165462 -1.238147
H -0.121745 -4.009407 -0.593703
H -2.744518 -1.241379 -2.549956
H -0.878482 0.348827 -2.822033
H 1.747743 -2.416234 -0.859509
N 3.554172 -0.254376 2.058022
H 4.090188 -0.037572 2.883616
N 1.650688 -0.027534 -1.898257
H 2.492276 -0.379829 -1.454427
C 4.076696 -1.163516 1.184003
C 1.604773 1.313787 -2.182527
O 0.561681 1.915189 -2.437968
O 3.575757 -1.394814 0.082049
C 5.345498 -1.919082 1.622911
C 6.422290 -1.627051 0.559076
H 6.055604 -1.894919 -0.433406
C 2.964161 2.038486 -2.305345
C 2.771116 3.497506 -1.863598
H 2.553299 3.552253 -0.793505
H 6.690627 -0.565260 0.550113
H 7.326919 -2.205284 0.771001
H 1.940424 3.953262 -2.403790
H 3.683006 4.070658 -2.059854
C 3.315380 1.995144 -3.810006
H 4.246572 2.541500 -3.993521
H 2.519987 2.454309 -4.402860
H 3.449927 0.964319 -4.152899
C 4.105166 1.405808 -1.494962
H 5.013475 2.001668 -1.632185
H 4.339215 0.386293 -1.813989
H 3.872285 1.385911 -0.428628
C 4.994994 -3.420503 1.609185
H 4.238658 -3.655839 2.365340
H 5.887477 -4.016529 1.823592
H 4.604325 -3.708379 0.631449
C 5.868014 -1.524095 3.012247
H 6.164876 -0.469562 3.055953
H 6.758259 -2.113554 3.249216
H 5.131792 -1.720824 3.799180

E = -1765.65915125 Hartree
ZPVE = 433.75481 kcal/mol
Zero-point correction= 0.691232 (Hartree/Particle)
Thermal correction to Energy= 0.730434
Thermal correction to Enthalpy= 0.731378
Thermal correction to Gibbs Free Energy= 0.618451
Sum of electronic and zero-point Energies= -1764.967919
Sum of electronic and thermal Energies= -1764.928717
Sum of electronic and thermal Enthalpies= -1764.927773
Sum of electronic and thermal Free Energies= -1765.040700

Conformer 2 without dispersion
N -6.767359 0.498401 0.345240
N -6.765860 -0.511775 -0.392812
C -5.569743 1.184111 0.742087
C -3.381256 2.744964 1.588705
C -5.441058 1.547226 2.087370
C -4.630840 1.649109 -0.189086
C -3.559067 2.427850 0.234872
C -4.339216 2.289818 2.504905
H -6.195525 1.221123 2.796656
H -4.745176 1.402564 -1.238997
H -2.837339 2.779894 -0.495871
H -4.229555 2.532612 3.559033
C -5.571975 -1.223488 -0.749798
C -3.390512 -2.825524 -1.537702
C -4.624288 -1.643150 0.195837
C -5.455058 -1.653793 -2.076075
C -4.356988 -2.417732 -2.466395
C -3.556162 -2.439756 -0.199196
H -4.730739 -1.351888 1.234723
H -6.218345 -1.364901 -2.792112
H -4.258134 -2.716120 -3.507182
H -2.824959 -2.754481 0.539217
C -2.214791 3.604484 2.058119
H -2.501127 4.662983 1.996740
H -2.044289 3.405380 3.123510
C -2.216031 -3.697915 -1.962112
H -2.123129 -3.650284 -3.053852
H -2.446035 -4.745217 -1.725191
C -0.921812 3.406240 1.290993
C 1.470977 3.014093 -0.165041
C -0.251359 2.175739 1.304990
C -0.360640 4.441680 0.536146
C 0.817363 4.253647 -0.181161
C 0.928704 1.966843 0.595362
H -0.665964 1.349617 1.876167
H -0.851131 5.410977 0.503512
H 1.228354 5.075237 -0.764065
H 1.421216 1.006800 0.619621

C -0.897916 -3.326513 -1.310596
C 1.511798 -2.577792 -0.020116
C -0.222084 -2.151250 -1.667697
C -0.329651 -4.118425 -0.308706
C 0.855448 -3.764828 0.338427
C 0.961753 -1.781713 -1.041832
H -0.633916 -1.509367 -2.442769
H -0.824163 -5.041967 -0.016113
H 1.272934 -4.388216 1.114852
H 1.467837 -0.865985 -1.331990
N 2.648554 2.902089 -0.941666
H 2.895166 3.747313 -1.435422
N 2.701823 -2.121333 0.586532
H 3.051371 -1.243167 0.219967
C 3.465795 1.828002 -1.166173
C 3.407591 -2.705677 1.616133
O 3.071768 -3.758929 2.146146
O 3.293607 0.729920 -0.634046
C 4.620675 2.094157 -2.166635
C 5.492917 0.832998 -2.261116
H 5.955541 0.597219 -1.299827
C 4.675419 -1.936621 2.070797
C 5.662931 -1.808700 0.889756
H 5.252057 -1.211194 0.071769
H 4.904268 -0.033873 -2.569299
H 6.289260 0.990897 -2.995120
H 5.932515 -2.792947 0.492909
H 6.583876 -1.320252 1.227859
C 5.335968 -2.735810 3.205371
H 6.238582 -2.217246 3.545873
H 5.613160 -3.738932 2.872680
H 4.657490 -2.850589 4.053988
C 4.286654 -0.533513 2.589952
H 5.174756 -0.028080 2.985779
H 3.554671 -0.604326 3.401292
H 3.868709 0.101917 1.804366
C 5.489898 3.278177 -1.684310
H 4.947654 4.230074 -1.670225
H 6.342802 3.406715 -2.358368
H 5.880497 3.099978 -0.677701
C 4.024544 2.406296 -3.559753
H 3.391533 1.585453 -3.910772
H 4.832519 2.542698 -4.285727
H 3.421734 3.320590 -3.565700

E = -1765.45783155 Hartree
ZPVE = 432.31339 kcal/mol
Zero-point correction= 0.688935 (Hartree/Particle)
Thermal correction to Energy= 0.728995
Thermal correction to Enthalpy= 0.729939
Thermal correction to Gibbs Free Energy= 0.612544

Sum of electronic and zero-point Energies= -1764.768896
Sum of electronic and thermal Energies= -1764.728837
Sum of electronic and thermal Enthalpies= -1764.727892
Sum of electronic and thermal Free Energies= -1764.845287

Conformer 2 with dispersion

N -4.995455 -4.247217 -0.349670
N -3.779242 -4.447535 -0.584203
C -5.421185 -2.937727 0.072532
C -6.047429 -0.283749 0.704273
C -4.909785 -2.358193 1.240274
C -6.353193 -2.245472 -0.698060
C -6.638294 -0.912960 -0.394685
C -5.227186 -1.044816 1.548208
H -4.221734 -2.919390 1.861608
H -6.781586 -2.724694 -1.572497
H -7.305857 -0.348406 -1.040020
H -4.781537 -0.580159 2.422725
C -2.845992 -3.349398 -0.554077
C -1.039470 -1.207879 -0.363715
C -1.716708 -3.457696 0.260586
C -3.017853 -2.213144 -1.352904
C -2.118255 -1.160075 -1.254320
C -0.836843 -2.382847 0.371026
H -1.567037 -4.363620 0.839209
H -3.868410 -2.144663 -2.019680
H -2.278198 -0.265343 -1.848460
H 0.017821 -2.452665 1.036606
C -6.157850 1.213702 0.918392
H -6.964048 1.619942 0.298924
H -6.407406 1.435594 1.961535
C -0.169420 0.015527 -0.171335
H -0.539159 0.580946 0.691323
H -0.316144 0.678172 -1.032309
C -4.834661 1.868406 0.562256
C -2.275551 2.853983 -0.114692
C -3.896133 2.190422 1.545686
C -4.470825 2.061973 -0.775299
C -3.214570 2.550414 -1.112382
C -2.628276 2.676426 1.229342
H -4.154844 2.055052 2.592430
H -5.173001 1.808804 -1.564609
H -2.951573 2.689963 -2.158492
H -1.910312 2.907913 2.001700
C 1.307410 -0.247114 0.014391
C 4.085456 -0.656956 0.300868
C 2.005934 0.316933 1.086832
C 2.026721 -1.023278 -0.902611
C 3.396982 -1.233816 -0.777632

C 3.375526 0.116329 1.229457
H 1.478128 0.945213 1.796761
H 1.500232 -1.481385 -1.735258
H 3.940984 -1.831079 -1.493949
H 3.899996 0.566369 2.069476
N -1.018296 3.338896 -0.527662
H -0.950935 3.561211 -1.509324
N 5.469114 -0.812134 0.512039
H 5.820167 -0.357125 1.340960
C 0.148118 3.427655 0.197259
C 6.396248 -1.481154 -0.253844
O 6.113606 -2.071939 -1.287548
O 0.216224 3.125557 1.383450
C 1.362086 3.949345 -0.603154
C 2.578976 3.983955 0.330713
H 2.382686 4.604005 1.208060
C 7.838961 -1.425597 0.300579
C 8.315845 0.039949 0.371424
H 7.732127 0.639629 1.077777
H 2.831833 2.980472 0.677411
H 3.440021 4.394424 -0.205953
H 8.252067 0.520792 -0.609257
H 9.359664 0.075196 0.699340
C 8.747527 -2.213063 -0.652218
H 9.777945 -2.190809 -0.283924
H 8.723674 -1.786749 -1.657353
H 8.424253 -3.253216 -0.730671
C 7.886429 -2.066458 1.703120
H 8.918861 -2.088302 2.066404
H 7.514221 -3.094878 1.677087
H 7.293647 -1.513816 2.439606
C 1.062115 5.375379 -1.111752
H 0.227423 5.402474 -1.819868
H 1.941094 5.775997 -1.626345
H 0.819557 6.045955 -0.281858
C 1.661058 3.013057 -1.793053
H 1.856808 1.993014 -1.453273
H 2.549076 3.369092 -2.324722
H 0.842540 2.980665 -2.520812

E = -1765.64073791 Hartree
ZPVE = 432.99025 kcal/mol
Zero-point correction= 0.690014 (Hartree/Particle)
Thermal correction to Energy= 0.729913
Thermal correction to Enthalpy= 0.730857
Thermal correction to Gibbs Free Energy= 0.614010
Sum of electronic and zero-point Energies= -1764.950724
Sum of electronic and thermal Energies= -1764.910825
Sum of electronic and thermal Enthalpies= -1764.909881
Sum of electronic and thermal Free Energies= -1765.026728

Compound 6 Boc
Conformer 0 without dispersion
H 2.331886 1.557380 -3.866465
H 3.592984 -0.389760 -3.614491
C 2.136718 1.596500 -2.797788
C 3.452578 -0.562479 -2.540099
H 2.860225 -1.483326 -2.460126
H 1.037828 3.455882 -2.915211
C 1.400118 2.657414 -2.275210
C 4.800884 -0.795290 -1.881010
C 2.651889 0.594844 -1.966137
H 5.555568 1.178240 -2.294912
C 5.777500 0.209501 -1.854810
C 5.119722 -2.023464 -1.293522
H 4.387868 -2.827426 -1.295190
C 7.027462 0.012173 -1.275465
C 6.363828 -2.242214 -0.707184
H 7.767292 0.800006 -1.267315
C 7.331570 -1.228549 -0.692067
C 1.110166 2.704261 -0.907177
H -1.454523 1.768816 -1.009490
C 2.403346 0.685936 -0.587646
H 6.585941 -3.207214 -0.257208
H -8.672668 -2.445646 -0.306622
N 8.566373 -1.520339 -0.082924
O 9.763524 0.440688 -0.334944
N 0.453676 3.887030 -0.430277
C -9.667713 -0.708489 -0.058017
N -8.566390 -1.520352 0.082951
H -2.825305 -0.060678 -0.079364
C 1.635023 1.716358 -0.058401
H -6.586001 -3.207266 0.257349
C 9.667718 -0.708496 0.057989
C -1.635024 1.716368 0.058429
H 2.825293 -0.060697 0.079379
H 8.672630 -2.445611 0.306707
C -6.363867 -2.242245 0.707268
O -9.763492 0.440722 0.334843
C -7.331583 -1.228556 0.692083
C -2.403353 0.685947 0.587666
O 10.623021 -1.417075 0.710570
N -0.453661 3.887029 0.430322
C -5.119759 -2.023491 1.293601
H -4.387925 -2.827471 1.295322
C -1.110159 2.704260 0.907213
C -7.027445 0.012194 1.275406
H 1.454522 1.768798 1.009518
H -7.767255 0.800046 1.267202
C -4.800893 -0.795289 1.881016
C -5.777482 0.209525 1.854747
C -2.651895 0.594845 1.966157

H -2.860235 -1.483327 2.460134
C -3.452588 -0.562479 2.540109
C -1.400110 2.657404 2.275246
H -5.555528 1.178286 2.294791
C -2.136717 1.596490 2.797816
H -1.037815 3.455865 2.915253
H -3.592998 -0.389766 3.614502
H -2.331885 1.557363 3.866492
O -10.623031 -1.417086 -0.710558
C -11.933454 -0.817733 -1.019628
C -12.655909 -1.959174 -1.739123
H -13.661594 -1.641848 -2.029610
H -12.111769 -2.253566 -2.641121
H -12.743438 -2.833177 -1.087335
C -12.658759 -0.446572 0.277672
H -12.730547 -1.318238 0.935660
H -12.137246 0.352719 0.804306
H -13.674936 -0.112039 0.045657
C -11.752159 0.384800 -1.951233
H -12.733007 0.752208 -2.269527
H -11.219876 1.193248 -1.449918
H -11.193152 0.091871 -2.845426
C 11.933460 -0.817735 1.019597
C 12.658769 -0.446670 -0.277728
H 12.137272 0.352601 -0.804409
H 13.674954 -0.112146 -0.045737
H 12.730534 -1.318378 -0.935663
C 11.752199 0.384859 1.951129
H 11.193196 0.091996 2.845347
H 12.733057 0.752268 2.269390
H 11.219925 1.193286 1.449770
C 12.655891 -1.959150 1.739158
H 13.661584 -1.641830 2.029622
H 12.111748 -2.253475 2.641175
H 12.743397 -2.833195 1.087422

E = -1915.92063182 Hartree
ZPVE = 437.26590 kcal/mol
Zero-point correction= 0.696828 (Hartree/Particle)
Thermal correction to Energy= 0.739320
Thermal correction to Enthalpy= 0.740264
Thermal correction to Gibbs Free Energy= 0.610519
Sum of electronic and zero-point Energies= -1915.223804
Sum of electronic and thermal Energies= -1915.181312
Sum of electronic and thermal Enthalpies= -1915.180367
Sum of electronic and thermal Free Energies= -1915.310113

Conformer 0 with dispersion
H 2.090469 1.573067 -3.919141
H 3.334029 -0.394430 -3.670231
C 1.947290 1.642861 -2.844332

C 3.200504 -0.550223 -2.592856
H 2.577137 -1.447630 -2.491636
H 0.905749 3.537027 -2.946809
C 1.275619 2.740218 -2.309890
C 4.541878 -0.809973 -1.939092
C 2.462725 0.641658 -2.015766
H 5.306840 1.160486 -2.332011
C 5.523104 0.188033 -1.898569
C 4.840787 -2.042123 -1.353633
H 4.099084 -2.836247 -1.363707
C 6.764731 -0.021701 -1.308730
C 6.076702 -2.273374 -0.756396
H 7.511293 0.758660 -1.286853
C 7.050810 -1.267540 -0.728763
C 1.057671 2.822145 -0.932431
H -1.456460 1.916703 -0.985628
C 2.290873 0.768636 -0.629281
H 6.287610 -3.240161 -0.305935
H -8.373566 -2.496615 -0.278837
N 8.275443 -1.569872 -0.108610
O 9.479145 0.387451 -0.342144
N 0.439129 4.019488 -0.445404
C -9.375988 -0.762707 -0.045919
N -8.275443 -1.569876 0.108553
H -2.722946 0.020441 -0.028498
C 1.588625 1.836022 -0.086894
H -6.287602 -3.240167 0.305776
C 9.375983 -0.762707 0.045911
C -1.588627 1.836014 0.086941
H 2.722951 0.020450 0.028478
H 8.373567 -2.496632 0.278730
C -6.076695 -2.273402 0.756286
O -9.479152 0.387430 0.342197
C -7.050807 -1.267571 0.728713
C -2.290870 0.768604 0.629288
O 10.321723 -1.474989 0.705030
N -0.439139 4.019470 0.445531
C -4.840776 -2.042178 1.353526
H -4.099069 -2.836299 1.363553
C -1.057675 2.822105 0.932514
C -6.764730 -0.021763 1.308745
H 1.456454 1.916669 0.985677
H -7.511296 0.758596 1.286915
C -4.541869 -0.810058 1.939049
C -5.523099 0.187946 1.898586
C -2.462717 0.641571 2.015769
H -2.577124 -1.447736 2.491553
C -3.200491 -0.550334 2.592815
C -1.275618 2.740125 2.309970
H -5.306837 1.160377 2.332078
C -1.947283 1.642744 2.844372

H -0.905749 3.536910 2.946919
H -3.334008 -0.394586 3.670197
H -2.090459 1.572908 3.919179
O -10.321728 -1.474958 -0.705071
C -11.624340 -0.870286 -1.023393
C -12.346948 -2.005192 -1.747481
H -13.348041 -1.680640 -2.044351
H -11.796886 -2.301002 -2.645012
H -12.443317 -2.877876 -1.095728
C -12.354939 -0.495954 0.267197
H -12.429620 -1.367029 0.925022
H -11.833928 0.303204 0.793489
H -13.368891 -0.161266 0.027753
C -11.428814 0.329940 -1.951189
H -12.405005 0.698739 -2.280561
H -10.900106 1.135294 -1.442261
H -10.859493 0.033391 -2.837190
C 11.624333 -0.870330 1.023386
C 12.354934 -0.495929 -0.267183
H 11.833923 0.303256 -0.793432
H 13.368885 -0.161252 -0.027718
H 12.429618 -1.366969 -0.925053
C 11.428802 0.329846 1.951246
H 10.859480 0.033249 2.837230
H 12.404990 0.698630 2.280639
H 10.900092 1.135225 1.442359
C 12.346943 -2.005273 1.747416
H 13.348033 -1.680734 2.044307
H 11.796878 -2.301133 2.644929
H 12.443316 -2.877922 1.095617

E = -1916.10149092 Hartree
ZPVE = 438.07728 kcal/mol
Zero-point correction= 0.698121 (Hartree/Particle)
Thermal correction to Energy= 0.740477
Thermal correction to Enthalpy= 0.741421
Thermal correction to Gibbs Free Energy= 0.611805
Sum of electronic and zero-point Energies= -1915.403370
Sum of electronic and thermal Energies= -1915.361014
Sum of electronic and thermal Enthalpies= -1915.360070
Sum of electronic and thermal Free Energies= -1915.489686

Conformer 1 without dispersion

N 7.058621 0.218399 -0.741358
N 6.777857 1.437585 -0.711158
C 6.063620 -0.809179 -0.623970
C 4.247425 -2.953238 -0.428339
C 4.912682 -0.834952 -1.426307
C 6.341098 -1.891958 0.215509

C 5.422484 -2.935645 0.331780
C 4.027234 -1.899592 -1.329153
H 4.717422 -0.022880 -2.117824
H 7.260618 -1.890068 0.792713
H 5.626957 -3.751304 1.020623
H 3.130277 -1.900027 -1.941561
C 5.477493 1.935146 -0.354155
C 3.055293 3.171703 0.386615
C 4.934638 2.956821 -1.144092
C 4.835002 1.569942 0.835977
C 3.644737 2.194419 1.199489
C 3.721407 3.540763 -0.791425
H 5.467232 3.271299 -2.036338
H 5.269902 0.809751 1.475116
H 3.161002 1.912158 2.131063
H 3.292914 4.309368 -1.429549
C 3.214604 -4.062503 -0.277354
H 3.132338 -4.618181 -1.219971
H 3.564957 -4.776885 0.475798
C 1.740999 3.815035 0.777509
H 1.780898 4.888396 0.556710
H 1.624771 3.739274 1.867099
C 1.849340 -3.518580 0.105291
C -0.596130 -2.265865 0.786278
C 1.572134 -3.125114 1.420294
C 0.855136 -3.307686 -0.856635
C -0.348999 -2.690756 -0.529232
C 0.371666 -2.513715 1.773480
H 2.321730 -3.283621 2.192113
H 1.024380 -3.622155 -1.883904
H -1.101134 -2.520768 -1.293734
H 0.180701 -2.210590 2.793235
C 0.494187 3.234085 0.117221
C -1.895775 2.178579 -0.969412
C -0.639209 4.039182 -0.054766
C 0.404664 1.896460 -0.282788
C -0.765032 1.365135 -0.826525
C -1.818300 3.524569 -0.583079
H -0.604840 5.086241 0.236116
H 1.266655 1.245523 -0.176289
H -0.799077 0.330198 -1.134851
H -2.685249 4.171077 -0.699583
N -1.809616 -1.595706 1.023848
H -2.328676 -1.309664 0.197417
N -3.120906 1.733861 -1.514529
H -3.809773 2.445604 -1.711743
C -2.255524 -1.062229 2.204936
C -3.546325 0.456300 -1.758160
O -2.900106 -0.566292 -1.558202
O -1.726611 -1.177937 3.298544
C -4.093836 0.363692 3.010410

C -4.569272 -0.591199 4.110787
H -3.723026 -1.038032 4.632178
H -5.181989 -1.389720 3.680634
H -5.183042 -0.043355 4.833369
C -5.288083 0.970100 2.268173
H -4.946480 1.605951 1.446190
H -5.888953 1.577047 2.951892
H -5.924716 0.182386 1.854402
C -3.177781 1.467047 3.551583
H -2.823282 2.100749 2.732552
H -2.316626 1.040951 4.066584
H -3.734235 2.095329 4.254783
O -3.407816 -0.383997 1.948902
O -4.794861 0.515461 -2.268074
C -5.536010 -0.703513 -2.652271
C -5.765257 -1.586899 -1.422918
H -6.426131 -2.417527 -1.689995
H -6.246378 -1.012175 -0.625883
H -4.828244 -1.994734 -1.044592
C -4.790289 -1.435295 -3.771772
H -4.608748 -0.759841 -4.613428
H -5.401055 -2.269121 -4.131819
H -3.835892 -1.827430 -3.420350
C -6.859571 -0.130678 -3.164254
H -6.689292 0.539243 -4.011757
H -7.369098 0.430547 -2.375908
H -7.516059 -0.942154 -3.490857

E = -1915.92793876 Hartree
ZPVE = 437.81801 kcal/mol
Zero-point correction= 0.697707 (Hartree/Particle)
Thermal correction to Energy= 0.739649
Thermal correction to Enthalpy= 0.740593
Thermal correction to Gibbs Free Energy= 0.618705
Sum of electronic and zero-point Energies= -1915.230231
Sum of electronic and thermal Energies= -1915.188290
Sum of electronic and thermal Enthalpies= -1915.187345
Sum of electronic and thermal Free Energies= -1915.309234

Conformer 1 with dispersion

N 7.002416 0.162113 -1.121342
N 6.656782 1.358278 -1.256068
C 6.070535 -0.838263 -0.686693
C 4.316558 -2.833417 0.219284
C 4.832943 -1.025114 -1.318279
C 6.462989 -1.699259 0.338658
C 5.574713 -2.668403 0.805090
C 3.974109 -2.015657 -0.867861
H 4.543710 -0.379198 -2.139005

H 7.441022 -1.570321 0.790910
H 5.863867 -3.303296 1.638147
H 3.003019 -2.135654 -1.337812
C 5.371052 1.829290 -0.821679
C 2.908798 2.923367 -0.028246
C 4.648278 2.643350 -1.700501
C 4.890115 1.608297 0.473654
C 3.673212 2.160102 0.860453
C 3.414506 3.155554 -1.314380
H 5.054489 2.841348 -2.687134
H 5.460616 1.001101 1.166132
H 3.302804 1.983877 1.866375
H 2.835368 3.751046 -2.014623
C 3.319608 -3.849199 0.749355
H 3.235322 -4.688058 0.047386
H 3.698995 -4.266475 1.688539
C 1.563811 3.476731 0.383101
H 1.526623 4.552427 0.175264
H 1.454467 3.372672 1.469821
C 1.954163 -3.228938 0.954880
C -0.514096 -1.886266 1.225585
C 1.732955 -2.316851 1.993348
C 0.906944 -3.467063 0.061075
C -0.311624 -2.807520 0.187269
C 0.519905 -1.652277 2.145533
H 2.537159 -2.107703 2.694143
H 1.047816 -4.170854 -0.755172
H -1.109478 -2.987481 -0.526132
H 0.363254 -0.949309 2.951032
C 0.380775 2.809136 -0.294993
C -1.909224 1.571407 -1.378449
C -0.712785 3.569279 -0.718565
C 0.323504 1.421900 -0.474112
C -0.794821 0.801233 -1.023994
C -1.847990 2.963410 -1.248087
H -0.689457 4.650295 -0.612106
H 1.166974 0.806061 -0.177518
H -0.810381 -0.269010 -1.159900
H -2.699315 3.571830 -1.543357
N -1.755550 -1.233057 1.257129
H -2.314727 -1.331041 0.413586
N -3.118035 1.022377 -1.858052
H -3.841709 1.686937 -2.091970
C -2.121231 -0.186772 2.058804
C -3.574209 -0.257401 -1.702972
O -2.891506 -1.226778 -1.390161
O -1.542684 0.197818 3.060976
C -3.932906 1.461167 2.226949
C -4.430589 0.999001 3.597354
H -3.591657 0.758038 4.250286
H -5.064976 0.113781 3.490746

H -5.023942 1.792609 4.061949
C -5.105474 1.762462 1.293647
H -4.741116 2.030764 0.299025
H -5.694166 2.595522 1.687537
H -5.758539 0.890903 1.196640
C -3.000826 2.670889 2.324929
H -2.600963 2.920872 1.339591
H -2.170311 2.468618 2.999459
H -3.564356 3.531402 2.699812
O -3.263907 0.347489 1.547207
O -4.893036 -0.280159 -1.969235
C -5.663431 -1.533895 -1.865114
C -5.570034 -2.095791 -0.445013
H -6.308708 -2.893916 -0.325321
H -5.785858 -1.314292 0.288204
H -4.580660 -2.500864 -0.238918
C -5.173374 -2.525354 -2.919962
H -5.228325 -2.077907 -3.916635
H -5.811106 -3.414338 -2.908755
H -4.145450 -2.828868 -2.721935
C -7.085152 -1.064874 -2.168388
H -7.135563 -0.604799 -3.158850
H -7.414031 -0.331507 -1.427034
H -7.771184 -1.915720 -2.144452

E = -1916.12937071 Hartree
ZPVE = 439.12885 kcal/mol
Zero-point correction= 0.699796 (Hartree/Particle)
Thermal correction to Energy= 0.741237
Thermal correction to Enthalpy= 0.742181
Thermal correction to Gibbs Free Energy= 0.622670
Sum of electronic and zero-point Energies= -1915.429574
Sum of electronic and thermal Energies= -1915.388134
Sum of electronic and thermal Enthalpies= -1915.387190
Sum of electronic and thermal Free Energies= -1915.506701

Conformer 2 without dispersion
N -6.973587 -0.625622 0.007439
N -6.973588 0.625621 -0.007400
C -5.788552 -1.426419 0.119910
C -3.649188 -3.241279 0.399599
C -4.793398 -1.203274 1.085162
C -5.737199 -2.585850 -0.661459
C -4.662138 -3.464918 -0.540075
C -3.746331 -2.106380 1.219877
H -4.847382 -0.331679 1.727749
H -6.539729 -2.778121 -1.366776
H -4.621101 -4.348106 -1.172475
H -2.978766 -1.919163 1.965798
C -5.788557 1.426418 -0.119893
C -3.649198 3.241277 -0.399622

C -5.737180 2.585841 0.661487
C -4.793431 1.203283 -1.085176
C -3.746366 2.106388 -1.219911
C -4.662121 3.464908 0.540083
H -6.539690 2.778105 1.366830
H -4.847435 0.331695 -1.727771
H -2.978822 1.919179 -1.965856
H -4.621064 4.348088 1.172492
C -2.471433 -4.195678 0.535340
H -2.425584 -4.573137 1.564111
H -2.643720 -5.066087 -0.107510
C -2.471440 4.195671 -0.535379
H -2.643724 5.066091 0.107458
H -2.425591 4.573114 -1.564156
C -1.141829 -3.549500 0.184644
C 1.259473 -2.214612 -0.483644
C -0.782168 -3.318261 -1.148839
C -0.256186 -3.120089 1.178526
C 0.925588 -2.459059 0.855257
C 0.399997 -2.669246 -1.495312
H -1.447247 -3.645974 -1.944026
H -0.492976 -3.295643 2.224897
H 1.585544 -2.115026 1.647003
H 0.657792 -2.501761 -2.531471
C -1.141839 3.549494 -0.184674
C 1.259460 2.214609 0.483634
C -0.256194 3.120072 -1.178549
C -0.782182 3.318266 1.148812
C 0.399982 2.669252 1.495295
C 0.925579 2.459044 -0.855270
H -0.492981 3.295616 -2.224923
H -1.447263 3.645987 1.943995
H 0.657772 2.501776 2.531456
H 1.585536 2.115002 -1.647012
N 2.449274 -1.497230 -0.732612
H 2.960384 -1.170424 0.080132
N 2.449260 1.497228 0.732612
H 2.960375 1.170420 -0.080127
C 2.902087 -1.020046 -1.934598
C 2.902064 1.020047 1.934603
O 2.486557 1.319465 3.039098
O 2.486587 -1.319459 -3.039097
C 4.779907 0.361770 -2.769040
C 5.452975 -0.828706 -3.458847
H 4.724682 -1.441563 -3.990502
H 5.973328 -1.450528 -2.723754
H 6.192801 -0.462226 -4.177208
C 5.810293 1.206501 -2.016659
H 5.325645 2.029251 -1.482660
H 6.529742 1.632979 -2.721584
H 6.355281 0.596725 -1.290679

C 3.978272 1.222902 -3.749713
H 3.508112 2.062763 -3.227884
H 3.206914 0.634039 -4.245625
H 4.651354 1.636451 -4.507632
O 3.902829 -0.120813 -1.681171
O 3.902808 0.120814 1.681186
C 4.779879 -0.361764 2.769062
C 3.978238 -1.222891 3.749735
H 4.651317 -1.636435 4.507660
H 3.508083 -2.062756 3.227908
H 3.206876 -0.634026 4.245638
C 5.452942 0.828716 3.458869
H 5.973299 1.450535 2.723775
H 6.192763 0.462241 4.177235
H 4.724646 1.441575 3.990516
C 5.810270 -1.206498 2.016693
H 6.355263 -0.596725 1.290713
H 5.325627 -2.029251 1.482695
H 6.529715 -1.632971 2.721624

E = -1915.92618315 Hartree
ZPVE = 437.93958 kcal/mol
Zero-point correction= 0.697901 (Hartree/Particle)
Thermal correction to Energy= 0.739707
Thermal correction to Enthalpy= 0.740651
Thermal correction to Gibbs Free Energy= 0.619752
Sum of electronic and zero-point Energies= -1915.228282
Sum of electronic and thermal Energies= -1915.186476
Sum of electronic and thermal Enthalpies= -1915.185532
Sum of electronic and thermal Free Energies= -1915.306431

Conformer 2 with dispersion
N -6.915053 -0.734234 0.544135
N -6.990054 0.514240 0.585042
C -5.692744 -1.448315 0.309875
C -3.469594 -3.141319 -0.028464
C -4.473502 -1.148395 0.938106
C -5.799006 -2.609087 -0.462196
C -4.689088 -3.427575 -0.651252
C -3.382410 -1.991758 0.769502
H -4.384818 -0.265436 1.559040
H -6.757292 -2.850625 -0.910423
H -4.778332 -4.317528 -1.268360
H -2.442420 -1.750793 1.256348
C -5.896813 1.360931 0.214402
C -3.843537 3.151782 -0.460455
C -5.590369 2.427588 1.063199
C -5.229112 1.231944 -1.010493
C -4.221476 2.128345 -1.340143
C -4.549732 3.294001 0.738309
H -6.152787 2.544852 1.983694

H -5.496663 0.429097 -1.687989
H -3.700533 2.018301 -2.286787
H -4.283550 4.093598 1.423913
C -2.296606 -4.097531 -0.159004
H -2.246350 -4.718203 0.744910
H -2.496255 -4.785993 -0.987502
C -2.634949 4.016202 -0.762887
H -2.707097 4.960435 -0.214008
H -2.609939 4.262169 -1.829902
C -0.955953 -3.428629 -0.365783
C 1.539880 -2.184280 -0.769686
C -0.605567 -2.891730 -1.609422
C -0.033126 -3.316203 0.678471
C 1.197533 -2.696287 0.487738
C 0.625822 -2.280364 -1.827716
H -1.311482 -2.957544 -2.432845
H -0.278827 -3.719067 1.657144
H 1.894755 -2.596147 1.314338
H 0.883854 -1.875625 -2.795633
C -1.364836 3.283429 -0.373955
C 0.840646 1.730664 0.425017
C -0.745796 2.404284 -1.269083
C -0.841187 3.381616 0.918028
C 0.253806 2.623428 1.328560
C 0.342515 1.633780 -0.882465
H -1.124515 2.311795 -2.282992
H -1.305294 4.058769 1.630220
H 0.650650 2.708003 2.330608
H 0.807212 0.952031 -1.589496
N 2.808478 -1.577602 -0.889047
H 3.397356 -1.597215 -0.065239
N 1.922267 0.892429 0.745375
H 2.163257 0.203460 0.049010
C 3.267940 -0.765930 -1.900141
C 2.623089 0.788997 1.911649
O 2.500467 1.484078 2.903439
O 2.656069 -0.479290 -2.915850
C 5.104966 0.822092 -2.247611
C 5.436137 0.431349 -3.687383
H 4.527344 0.238028 -4.256363
H 6.062240 -0.465602 -3.700882
H 5.991196 1.243328 -4.167110
C 6.375821 1.059924 -1.434648
H 6.125676 1.306471 -0.399127
H 6.943898 1.890342 -1.862437
H 7.007581 0.167478 -1.438303
C 4.171905 2.031382 -2.163187
H 3.891342 2.221050 -1.122919
H 3.266483 1.875242 -2.749515
H 4.687403 2.916836 -2.546316
O 4.492651 -0.319688 -1.544894

O 3.504528 -0.247850 1.767386
C 4.543664 -0.501663 2.774044
C 3.896190 -0.934979 4.089152
H 4.672287 -1.233790 4.800411
H 3.238392 -1.793414 3.921955
H 3.312962 -0.121418 4.519514
C 5.424470 0.737648 2.938654
H 5.837485 1.038784 1.971243
H 6.258604 0.506040 3.607579
H 4.856676 1.567976 3.357461
C 5.335438 -1.646575 2.142313
H 5.717841 -1.348902 1.161570
H 4.704914 -2.533178 2.019931
H 6.181047 -1.918036 2.779809

E = -1916.12312992 Hartree
ZPVE = 438.82188 kcal/mol
Zero-point correction= 0.699307 (Hartree/Particle)
Thermal correction to Energy= 0.741012
Thermal correction to Enthalpy= 0.741956
Thermal correction to Gibbs Free Energy= 0.621652
Sum of electronic and zero-point Energies= -1915.423823
Sum of electronic and thermal Energies= -1915.382118
Sum of electronic and thermal Enthalpies= -1915.381174
Sum of electronic and thermal Free Energies= -1915.501478

Conformer 3 without dispersion
N -7.251025 0.099530 -0.371785
N -7.062954 1.214599 0.163534
C -6.204289 -0.856441 -0.589195
C -4.311736 -2.884855 -1.073715
C -5.342092 -1.276321 0.435300
C -6.165258 -1.501627 -1.829189
C -5.204711 -2.482868 -2.073667
C -4.417221 -2.282791 0.190092
H -5.399472 -0.811720 1.413570
H -6.871560 -1.206686 -2.599067
H -5.154942 -2.947861 -3.055059
H -3.741837 -2.589346 0.983798
C -5.765131 1.766070 0.435056
C -3.380983 3.168202 0.994847
C -5.619405 2.463864 1.639787
C -4.726330 1.803853 -0.508185
C -3.557398 2.503398 -0.227484
C -4.428447 3.127480 1.924550
H -6.444185 2.468529 2.345736
H -4.841024 1.298953 -1.460632
H -2.762200 2.526345 -0.966783
H -4.320865 3.644527 2.874837
C -3.238529 -3.930379 -1.339658
H -3.465638 -4.847519 -0.781877

H -3.264188 -4.203237 -2.401543
C -2.122502 3.977287 1.284912
H -2.093473 4.200391 2.357735
H -2.207377 4.948534 0.779364
C -1.852518 -3.445660 -0.951525
C 0.631452 -2.401345 -0.104533
C -1.125059 -4.045477 0.079568
C -1.289085 -2.326275 -1.580451
C -0.068467 -1.810258 -1.168915
C 0.106637 -3.545347 0.507685
H -1.530729 -4.922769 0.577697
H -1.828407 -1.833117 -2.384618
H 0.335335 -0.924763 -1.653596
H 0.646573 -4.017069 1.316438
C -0.818152 3.326489 0.865506
C 1.609585 2.106763 0.069874
C -0.233304 2.312023 1.635794
C -0.154767 3.712762 -0.303439
C 1.040289 3.119521 -0.712970
C 0.960516 1.712403 1.252006
H -0.718992 1.983608 2.551044
H -0.575299 4.506570 -0.916242
H 1.531133 3.433863 -1.622726
H 1.395676 0.928754 1.865660
N 1.827307 -1.770802 0.300308
H 2.074723 -0.929599 -0.203439
N 2.812777 1.440108 -0.260552
H 3.202438 0.821543 0.443786
C 2.591111 -2.046557 1.404455
C 3.596152 1.625053 -1.379234
O 3.346699 2.370668 -2.309629
O 2.496409 -3.027376 2.118478
C 4.560061 -1.121405 2.588893
C 3.961942 -1.209637 3.995902
H 3.416808 -2.142305 4.136263
H 3.282142 -0.370451 4.173930
H 4.767226 -1.153086 4.735418
C 5.315521 0.196322 2.402652
H 5.656194 0.304947 1.369541
H 6.186370 0.222272 3.063857
H 4.677430 1.050435 2.650614
C 5.457800 -2.316757 2.257663
H 5.841979 -2.233160 1.236414
H 4.914690 -3.256835 2.358484
H 6.311883 -2.331911 2.941885
O 3.479379 -1.020768 1.580409
O 4.682433 0.818013 -1.273438
C 5.691400 0.766050 -2.349729
C 6.352426 2.137885 -2.514839
H 7.181146 2.060705 -3.225874
H 6.757764 2.481821 -1.558134

H 5.640425 2.876061 -2.883723
C 5.047023 0.267283 -3.647133
H 4.550973 -0.694068 -3.480402
H 5.821774 0.120589 -4.406316
H 4.317155 0.983169 -4.024934
C 6.694296 -0.259732 -1.816777
H 6.205643 -1.222204 -1.639963
H 7.139605 0.082514 -0.878034
H 7.498037 -0.408614 -2.543622

E = -1915.92618048 Hartree
ZPVE = 437.89251 kcal/mol
Zero-point correction= 0.697826 (Hartree/Particle)
Thermal correction to Energy= 0.739687
Thermal correction to Enthalpy= 0.740631
Thermal correction to Gibbs Free Energy= 0.619392
Sum of electronic and zero-point Energies= -1915.228354
Sum of electronic and thermal Energies= -1915.186494
Sum of electronic and thermal Enthalpies= -1915.185549
Sum of electronic and thermal Free Energies= -1915.306789

Conformer 3 with dispersion
N -7.288791 -0.089311 -0.400790
N -7.122576 1.068976 0.042474
C -6.213295 -1.027340 -0.517081
C -4.210127 -2.969965 -0.817583
C -5.361228 -1.328318 0.555485
C -6.107911 -1.744487 -1.709988
C -5.090826 -2.685617 -1.864502
C -4.377872 -2.293796 0.400521
H -5.467174 -0.794707 1.493053
H -6.802777 -1.530784 -2.515535
H -4.983087 -3.208685 -2.810727
H -3.701968 -2.508752 1.222822
C -5.831548 1.640218 0.297511
C -3.463179 3.072723 0.811748
C -5.730332 2.455431 1.429058
C -4.751096 1.563994 -0.594585
C -3.587229 2.278466 -0.336216
C -4.546945 3.137294 1.695019
H -6.585989 2.536595 2.091506
H -4.828035 0.956107 -1.487884
H -2.756384 2.214780 -1.031703
H -4.472705 3.752892 2.587442
C -3.071106 -3.954821 -0.985583
H -3.243204 -4.839292 -0.360455
H -3.055936 -4.310650 -2.022906
C -2.224582 3.917015 1.063827
H -2.202327 4.196059 2.122879
H -2.327835 4.857613 0.506880

C -1.732787 -3.344059 -0.618635
C 0.704211 -2.144180 0.121386
C -0.880014 -3.949502 0.305478
C -1.337221 -2.120372 -1.175728
C -0.138470 -1.524352 -0.814298
C 0.334150 -3.371750 0.678686
H -1.167990 -4.896606 0.754110
H -1.992983 -1.615853 -1.878984
H 0.140707 -0.561327 -1.234008
H 0.980846 -3.848406 1.401360
C -0.912747 3.269768 0.682345
C 1.547470 2.084134 -0.025180
C -0.264235 2.386925 1.554075
C -0.308304 3.529868 -0.550689
C 0.904563 2.948602 -0.918787
C 0.949579 1.803484 1.213787
H -0.713725 2.156186 2.515834
H -0.789044 4.216205 -1.242884
H 1.357702 3.161640 -1.876029
H 1.443565 1.126286 1.903726
N 1.890038 -1.462360 0.452140
H 2.067764 -0.612271 -0.065784
N 2.782612 1.459136 -0.295748
H 3.232715 0.973286 0.474514
C 2.734406 -1.703799 1.499433
C 3.478493 1.442908 -1.481729
O 3.141035 1.985483 -2.518403
O 2.736283 -2.682622 2.221807
C 4.752383 -0.683234 2.508432
C 4.285929 -0.776198 3.960716
H 3.786528 -1.725462 4.150489
H 3.594971 0.041271 4.187947
H 5.149704 -0.686233 4.626554
C 5.431834 0.657698 2.233238
H 5.677747 0.751673 1.172539
H 6.351952 0.736098 2.818439
H 4.777088 1.488012 2.515620
C 5.657496 -1.846900 2.105769
H 5.932466 -1.761869 1.050852
H 5.162247 -2.803616 2.271445
H 6.574338 -1.817677 2.701927
O 3.582795 -0.636524 1.611074
O 4.594380 0.696080 -1.294380
C 5.504736 0.416110 -2.415804
C 6.121934 1.719995 -2.922112
H 6.884724 1.495410 -3.673810
H 6.602386 2.254942 -2.097441
H 5.363833 2.362676 -3.368702
C 4.756856 -0.344123 -3.512252
H 4.280287 -1.235809 -3.093728
H 5.465632 -0.665520 -4.281267

H 3.995079 0.282767 -3.974599
C 6.563300 -0.474767 -1.767872
H 6.105704 -1.383441 -1.367200
H 7.066344 0.051895 -0.952094
H 7.314197 -0.762166 -2.508854

E = -1916.12337394 Hartree
ZPVE = 439.03122 kcal/mol
Zero-point correction= 0.699641 (Hartree/Particle)
Thermal correction to Energy= 0.741202
Thermal correction to Enthalpy= 0.742147
Thermal correction to Gibbs Free Energy= 0.621779
Sum of electronic and zero-point Energies= -1915.423733
Sum of electronic and thermal Energies= -1915.382171
Sum of electronic and thermal Enthalpies= -1915.381227
Sum of electronic and thermal Free Energies= -1915.501595

Conformer 4 without dispersion
N -7.251022 0.099520 -0.371800
N -7.062955 1.214591 0.163518
C -6.204283 -0.856450 -0.589202
C -4.311726 -2.884864 -1.073705
C -6.165245 -1.501639 -1.829194
C -5.342091 -1.276326 0.435299
C -4.417218 -2.282796 0.190099
C -5.204696 -2.482880 -2.073664
H -6.871541 -1.206700 -2.599076
H -5.399477 -0.811722 1.413567
H -3.741840 -2.589349 0.983811
H -5.154921 -2.947875 -3.055054
C -5.765135 1.766064 0.435048
C -3.380991 3.168200 0.994848
C -5.619418 2.463864 1.639777
C -4.726328 1.803844 -0.508187
C -3.557397 2.503390 -0.227481
C -4.428462 3.127482 1.924545
H -6.444202 2.468532 2.345721
H -4.841015 1.298939 -1.460632
H -2.762195 2.526334 -0.966775
H -4.320886 3.644534 2.874829
C -3.238519 -3.930389 -1.339639
H -3.465627 -4.847525 -0.781851
H -3.264175 -4.203256 -2.401522
C -2.122512 3.977286 1.284918
H -2.093484 4.200385 2.357743
H -2.207387 4.948534 0.779374
C -1.852508 -3.445666 -0.951507
C 0.631462 -2.401346 -0.104524
C -1.125047 -4.045478 0.079587
C -1.289076 -2.326283 -1.580440
C -0.068459 -1.810264 -1.168907

C 0.106649 -3.545345 0.507700
H -1.530716 -4.922768 0.577720
H -1.828401 -1.833129 -2.384607
H 0.335342 -0.924771 -1.653592
H 0.646587 -4.017063 1.316455
C -0.818160 3.326491 0.865512
C 1.609578 2.106769 0.069877
C -0.233313 2.312020 1.635794
C -0.154773 3.712771 -0.303430
C 1.040284 3.119532 -0.712962
C 0.960507 1.712403 1.252006
H -0.719003 1.983599 2.551042
H -0.575304 4.506582 -0.916228
H 1.531129 3.433879 -1.622715
H 1.395665 0.928749 1.865655
N 1.827318 -1.770801 0.300313
H 2.074733 -0.929600 -0.203440
N 2.812769 1.440115 -0.260552
H 3.202433 0.821550 0.443785
C 2.591123 -2.046549 1.404460
C 3.596141 1.625057 -1.379238
O 3.346685 2.370672 -2.309632
O 2.496423 -3.027365 2.118488
C 4.560075 -1.121390 2.588889
C 3.961961 -1.209616 3.995900
H 3.416830 -2.142285 4.136267
H 3.282160 -0.370430 4.173926
H 4.767247 -1.153059 4.735413
C 5.315533 0.196338 2.402639
H 5.656200 0.304960 1.369526
H 6.186386 0.222291 3.063840
H 4.677443 1.050451 2.650602
C 5.457815 -2.316742 2.257660
H 5.841992 -2.233147 1.236411
H 4.914705 -3.256820 2.358486
H 6.311899 -2.331893 2.941882
O 3.479390 -1.020758 1.580408
O 4.682420 0.818014 -1.273445
C 5.691380 0.766045 -2.349742
C 6.352412 2.137877 -2.514858
H 7.181126 2.060692 -3.225899
H 6.757757 2.481812 -1.558157
H 5.640411 2.876055 -2.883739
C 5.046994 0.267278 -3.647141
H 4.550939 -0.694070 -3.480406
H 5.821740 0.120578 -4.406328
H 4.317127 0.983168 -4.024940
C 6.694274 -0.259741 -1.816794
H 6.205618 -1.222210 -1.639974
H 7.139591 0.082505 -0.878054
H 7.498010 -0.408628 -2.543643

E = -1915.92618048 Hartree
ZPVE = 437.89251 kcal/mol
Zero-point correction= 0.697826 (Hartree/Particle)
Thermal correction to Energy= 0.739687
Thermal correction to Enthalpy= 0.740631
Thermal correction to Gibbs Free Energy= 0.619392
Sum of electronic and zero-point Energies= -1915.228354
Sum of electronic and thermal Energies= -1915.186494
Sum of electronic and thermal Enthalpies= -1915.185549
Sum of electronic and thermal Free Energies= -1915.306789

Conformer 4 with dispersion
N -7.129093 -0.497502 -0.060420
N -7.047389 0.733916 0.146287
C -5.980510 -1.351712 -0.094139
C -3.845488 -3.173112 -0.189856
C -5.903044 -2.286631 -1.128482
C -5.023616 -1.366737 0.930874
C -3.975060 -2.273415 0.878928
C -4.824050 -3.167119 -1.187830
H -6.674071 -2.290900 -1.892018
H -5.102399 -0.662694 1.751304
H -3.222800 -2.268638 1.661742
H -4.746002 -3.864401 -2.017525
C -5.805164 1.450286 0.175228
C -3.559254 3.147786 0.197508
C -5.704579 2.476847 1.118932
C -4.789288 1.291521 -0.779881
C -3.686533 2.136210 -0.764740
C -4.578307 3.294607 1.144828
H -6.514572 2.614895 1.827736
H -4.869361 0.516428 -1.532322
H -2.904755 2.003586 -1.505788
H -4.502200 4.077332 1.894720
C -2.656970 -4.114674 -0.253632
H -2.770068 -4.908580 0.494719
H -2.647768 -4.611264 -1.230850
C -2.386251 4.114759 0.175490
H -2.361830 4.657890 1.126100
H -2.570090 4.868886 -0.600834
C -1.349320 -3.389877 -0.015338
C 0.981991 -1.904413 0.504588
C -0.559034 -3.654085 1.104279
C -0.937991 -2.372887 -0.887684
C 0.212284 -1.639459 -0.639617
C 0.602407 -2.929324 1.375360
H -0.858575 -4.439605 1.793055
H -1.542923 -2.137810 -1.758683
H 0.512156 -0.844540 -1.318235
H 1.200475 -3.136694 2.251409

C -1.041632 3.471240 -0.078869
C 1.446926 2.243167 -0.563756
C -0.301833 2.911879 0.969388
C -0.507767 3.398403 -1.369150
C 0.720178 2.791339 -1.625643
C 0.927897 2.307303 0.737170
H -0.695869 2.946096 1.981213
H -1.058582 3.836191 -2.197431
H 1.113859 2.735013 -2.630456
H 1.490073 1.871616 1.557868
N 2.107214 -1.085562 0.705458
H 2.236184 -0.350081 0.026207
N 2.703743 1.623300 -0.733993
H 3.255800 1.456880 0.100785
C 2.920962 -1.005996 1.800414
C 3.161746 0.998144 -1.871313
O 2.589605 0.972083 -2.946629
O 2.946873 -1.769559 2.747368
C 4.835125 0.365883 2.559861
C 4.301961 0.659236 3.961698
H 3.827027 -0.223623 4.388680
H 3.572254 1.473897 3.925740
H 5.127022 0.969836 4.609953
C 5.471376 1.607510 1.936111
H 5.760865 1.406537 0.900875
H 6.361446 1.897768 2.500593
H 4.771604 2.449093 1.947755
C 5.809864 -0.811550 2.539830
H 6.145454 -1.007387 1.517603
H 5.346118 -1.711220 2.943604
H 6.687515 -0.565668 3.144973
O 3.709803 0.100408 1.648398
O 4.332777 0.392371 -1.565664
C 4.921410 -0.598925 -2.481675
C 5.368928 0.086673 -3.772038
H 5.904876 -0.630987 -4.400741
H 6.046677 0.914623 -3.544036
H 4.511920 0.471430 -4.324094
C 3.917032 -1.726149 -2.730036
H 3.565483 -2.135574 -1.778086
H 4.403341 -2.530975 -3.289006
H 3.059980 -1.371694 -3.302476
C 6.118646 -1.110019 -1.682888
H 5.784076 -1.567797 -0.748174
H 6.801999 -0.289747 -1.445894
H 6.663622 -1.859874 -2.262623

E = -1916.12342624 Hartree
ZPVE = 438.88915 kcal/mol
Zero-point correction= 0.699414 (Hartree/Particle)
Thermal correction to Energy= 0.741089

Thermal correction to Enthalpy= 0.742033
Thermal correction to Gibbs Free Energy= 0.621381
Sum of electronic and zero-point Energies= -1915.424012
Sum of electronic and thermal Energies= -1915.382337
Sum of electronic and thermal Enthalpies= -1915.381393
Sum of electronic and thermal Free Energies= -1915.502046

Compound 7 H

Conformer 0 without dispersion
H 3.088064 1.229657 -3.299668
H 4.272123 -0.716354 -2.792132
C 2.673775 1.266831 -2.295275
C 3.909885 -0.892292 -1.771300
H 3.313363 -1.812674 -1.821026
H 1.623656 3.127177 -2.635814
C 1.844123 2.327067 -1.935983
C 5.086023 -1.128463 -0.840246
C 3.004390 0.263195 -1.376633
H 5.938590 0.832822 -1.111718
C 6.047924 -0.131449 -0.621707
C 5.259286 -2.348197 -0.178230
H 4.531975 -3.143526 -0.324532
C 7.136159 -0.342921 0.216428
C 6.344557 -2.574374 0.666798
H 7.869054 0.447013 0.362474
C 7.303950 -1.572800 0.877801
C 1.273964 2.371198 -0.658945
H -1.208375 1.429966 -1.284612
C 2.472516 0.351849 -0.080502
H 6.452884 -3.535117 1.164382
H -8.309596 -2.528854 -2.374235
N 8.427319 -1.807673 1.675499
N 0.532678 3.553604 -0.327783
N -8.427297 -1.807723 -1.675499
H -2.746901 -0.396357 -0.657902
C 1.609550 1.380674 0.278371
H -6.452854 -3.535144 -1.164330
C -1.609558 1.380681 -0.278383
H 2.746882 -0.396365 0.657917
H 8.309632 -2.528792 2.374250
C -6.344539 -2.574390 -0.666763
C -7.303936 -1.572826 -0.877795
C -2.472530 0.351866 0.080506
N -0.532671 3.553612 0.327740
C -5.259277 -2.348190 0.178270
H -4.531962 -3.143511 0.324596
C -1.273965 2.371217 0.658919
C -7.136159 -0.342934 -0.216445
H 1.208367 1.429975 1.284599
H -7.869058 0.446992 -0.362513
C -5.086029 -1.128442 0.840264

C -6.047934 -0.131438 0.621696
C -3.004403 0.263233 1.376639
H -3.313379 -1.812625 1.821082
C -3.909902 -0.892244 1.771325
C -1.844124 2.327107 1.935959
H -5.938611 0.832843 1.111690
C -2.673782 1.266880 2.295266
H -1.623651 3.127225 2.635778
H -4.272150 -0.716281 2.792148
H -3.088070 1.229724 3.299660
H -8.851763 -0.977817 -2.066955
H 8.851785 -0.977757 2.066934

E = -1224.22197570 Hartree
ZPVE = 277.30597 kcal/mol
Zero-point correction= 0.441915 (Hartree/Particle)
Thermal correction to Energy= 0.467882
Thermal correction to Enthalpy= 0.468826
Thermal correction to Gibbs Free Energy= 0.379279
Sum of electronic and zero-point Energies= -1223.780061
Sum of electronic and thermal Energies= -1223.754094
Sum of electronic and thermal Enthalpies= -1223.753150
Sum of electronic and thermal Free Energies= -1223.842696

Conformer 0 with dispersion
H 2.915143 1.218003 -3.355948
H 4.072926 -0.749499 -2.837112
C 2.534737 1.286305 -2.340397
C 3.701541 -0.907637 -1.817167
H 3.070081 -1.804100 -1.861326
H 1.543134 3.181553 -2.670363
C 1.760589 2.383325 -1.968181
C 4.857976 -1.170990 -0.875438
C 2.851662 0.283275 -1.419400
H 5.724438 0.784590 -1.117249
C 5.820259 -0.182344 -0.630765
C 4.999300 -2.392676 -0.213268
H 4.264351 -3.176643 -0.377826
C 6.885155 -0.406316 0.231888
C 6.061044 -2.631345 0.656772
H 7.621070 0.375596 0.400876
C 7.023897 -1.639712 0.892159
C 1.238748 2.463071 -0.674738
H -1.195360 1.552490 -1.281601
C 2.372805 0.408393 -0.106597
H 6.147985 -3.592589 1.156631
H -7.979030 -2.607219 -2.411209
N 8.124039 -1.886934 1.716881
N 0.527064 3.660102 -0.337174
N -8.124010 -1.886973 -1.716892
H -2.647207 -0.341458 -0.629258

C 1.566161 1.474582 0.266255
H -6.147962 -3.592617 -1.156579
C -1.566161 1.474575 -0.266259
H 2.647207 -0.341446 0.629281
H 7.979070 -2.607165 2.411214
C -6.061029 -2.631362 -0.656740
C -7.023879 -1.639734 -0.892160
C -2.372809 0.408394 0.106608
N -0.527068 3.660107 0.337137
C -4.999297 -2.392674 0.213310
H -4.264350 -3.176638 0.377894
C -1.238753 2.463081 0.674717
C -6.885146 -0.406325 -0.231913
H 1.195366 1.552517 1.281598
H -7.621060 0.375584 -0.400927
C -4.857983 -1.170975 0.875457
C -5.820262 -0.182334 0.630750
C -2.851674 0.283302 1.419410
H -3.070101 -1.804063 1.861383
C -3.701560 -0.907601 1.817194
C -1.760601 2.383359 1.968159
H -5.724448 0.784610 1.117215
C -2.534754 1.286349 2.340391
H -1.543150 3.181601 2.670327
H -4.072957 -0.749438 2.837131
H -2.915167 1.218066 3.355940
H -8.543240 -1.060911 -2.121562
H 8.543274 -1.060862 2.121528

E = -1224.34549915 Hartree
ZPVE = 277.79401 kcal/mol
Zero-point correction= 0.442693 (Hartree/Particle)
Thermal correction to Energy= 0.468586
Thermal correction to Enthalpy= 0.469530
Thermal correction to Gibbs Free Energy= 0.380075
Sum of electronic and zero-point Energies= -1223.902806
Sum of electronic and thermal Energies= -1223.876913
Sum of electronic and thermal Enthalpies= -1223.875969
Sum of electronic and thermal Free Energies= -1223.965425

Conformer 1 without dispersion
N 4.861842 0.335377 -0.185733
N 4.746564 -0.902694 -0.326855
C 3.739824 1.221951 -0.074471
C 1.678799 3.131632 0.138496
C 2.692049 1.237025 -1.007630
C 3.789988 2.204001 0.919864
C 2.749891 3.124342 1.040605
C 1.684655 2.188023 -0.900827
H 2.672088 0.505612 -1.808315
H 4.630076 2.215558 1.607501

H 2.775747 3.855461 1.844765
H 0.869406 2.185167 -1.618370
C 3.503216 -1.608882 -0.192240
C 1.245174 -3.272981 0.099116
C 2.621382 -1.424410 0.885544
C 3.275970 -2.657295 -1.089907
C 2.143086 -3.459087 -0.958411
C 1.514583 -2.252491 1.024110
H 2.810546 -0.643025 1.612561
H 3.990830 -2.826585 -1.889195
H 1.967852 -4.256848 -1.675851
H 0.837923 -2.099137 1.860586
C 0.549705 4.140767 0.267465
H 0.651238 4.651802 1.234265
H 0.673963 4.922696 -0.493743
C 0.022599 -4.162057 0.262812
H 0.074870 -4.673343 1.231847
H 0.055639 -4.949249 -0.499682
C -0.842985 3.548808 0.130443
C -3.447444 2.433114 -0.128048
C -1.798272 4.121703 -0.715189
C -1.223251 2.407550 0.852865
C -2.492546 1.856664 0.731321
C -3.077541 3.583167 -0.844561
H -1.538470 5.005707 -1.293635
H -0.503164 1.929170 1.511655
H -2.754081 0.965341 1.295046
H -3.795000 4.049997 -1.515753
C -1.301495 -3.420757 0.173911
C -3.773080 -2.046447 -0.009214
C -2.128647 -3.265594 1.290372
C -1.736386 -2.866509 -1.038546
C -2.947888 -2.191699 -1.135210
C -3.346312 -2.588986 1.208431
H -1.825747 -3.690136 2.244412
H -1.113112 -2.962482 -1.923913
H -3.258364 -1.767576 -2.086913
H -3.970705 -2.488726 2.093049
N -4.729839 1.895174 -0.221948
H -4.790178 0.890647 -0.064426
N -4.981332 -1.317432 -0.098608
H -5.643613 -1.555946 0.630949
H -5.428334 -1.405952 -1.004369
H -5.240505 2.179544 -1.047076

E = -1224.22724177 Hartree
ZPVE = 278.01172 kcal/mol
Zero-point correction= 0.443040 (Hartree/Particle)
Thermal correction to Energy= 0.468353
Thermal correction to Enthalpy= 0.469297
Thermal correction to Gibbs Free Energy= 0.385315

Sum of electronic and zero-point Energies= -1223.784202
Sum of electronic and thermal Energies= -1223.758889
Sum of electronic and thermal Enthalpies= -1223.757944
Sum of electronic and thermal Free Energies= -1223.841927

Conformer 1 with dispersion
N 4.897894 0.561953 -0.273353
N 4.850050 -0.687085 -0.333719
C 3.715783 1.365556 -0.170464
C 1.495146 3.072493 0.053623
C 2.645597 1.248020 -1.068656
C 3.702931 2.373001 0.795578
C 2.583556 3.192128 0.923874
C 1.556961 2.102492 -0.958187
H 2.671795 0.485938 -1.839331
H 4.555528 2.478194 1.458598
H 2.558265 3.944891 1.706932
H 0.722826 2.002866 -1.645786
C 3.637721 -1.426203 -0.126633
C 1.414300 -3.097797 0.277726
C 2.775504 -1.202061 0.958563
C 3.411722 -2.512469 -0.974370
C 2.292007 -3.320640 -0.787147
C 1.682538 -2.034772 1.152806
H 2.965269 -0.381725 1.639993
H 4.112105 -2.701202 -1.781347
H 2.106469 -4.147133 -1.467502
H 1.013658 -1.854563 1.989721
C 0.296557 3.986186 0.183971
H 0.390222 4.545292 1.124558
H 0.334559 4.743559 -0.610666
C 0.194817 -3.965383 0.482048
H 0.189431 -4.358278 1.505827
H 0.268149 -4.841003 -0.175872
C -1.056173 3.300402 0.128358
C -3.638985 2.115402 0.121602
C -2.160067 3.967630 -0.412327
C -1.269222 2.019823 0.655625
C -2.529423 1.432030 0.652317
C -3.428134 3.395987 -0.416637
H -2.026452 4.958977 -0.839252
H -0.431396 1.464395 1.066460
H -2.664134 0.436419 1.063217
H -4.266813 3.940816 -0.843506
C -1.125031 -3.256111 0.226826
C -3.627211 -2.066607 -0.332686
C -2.273471 -3.622577 0.934811
C -1.248283 -2.259423 -0.748462
C -2.476416 -1.668073 -1.025297
C -3.510165 -3.042767 0.663205

H -2.204781 -4.384147 1.707295
H -0.367943 -1.934633 -1.293683
H -2.544520 -0.880849 -1.770662
H -4.388970 -3.347770 1.225532
N -4.910810 1.553565 0.172692
H -4.939638 0.535974 0.138556
N -4.867992 -1.441309 -0.590420
H -5.661268 -2.006381 -0.310351
H -4.971633 -1.163541 -1.559812
H -5.580054 1.976431 -0.455718

E = -1224.35746558 Hartree
ZPVE = 278.61596 kcal/mol
Zero-point correction= 0.444003 (Hartree/Particle)
Thermal correction to Energy= 0.469189
Thermal correction to Enthalpy= 0.470133
Thermal correction to Gibbs Free Energy= 0.386224
Sum of electronic and zero-point Energies= -1223.913463
Sum of electronic and thermal Energies= -1223.888277
Sum of electronic and thermal Enthalpies= -1223.887333
Sum of electronic and thermal Free Energies= -1223.971242

Conformer 2 without dispersion
N 0.587664 4.088989 -0.213559
N -0.587659 4.088985 0.213583
C 1.307583 2.906491 -0.588800
C 2.949341 0.801127 -1.495786
C 0.786262 1.924055 -1.440663
C 2.661635 2.855025 -0.227863
C 3.459309 1.797795 -0.649700
C 1.608610 0.892924 -1.890283
H -0.249682 1.973835 -1.757179
H 3.067086 3.648856 0.391990
H 4.497114 1.747498 -0.332528
H 1.198441 0.142692 -2.561900
C -1.307572 2.906480 0.588814
C -2.949322 0.801103 1.495786
C -2.661625 2.855013 0.227879
C -0.786246 1.924038 1.440667
C -1.608590 0.892901 1.890280
C -3.459294 1.797776 0.649709
H -3.067080 3.648847 -0.391965
H 0.249699 1.973819 1.757182
H -1.198418 0.142665 2.561890
H -4.497100 1.747480 0.332539
C 3.839058 -0.321599 -2.005165
H 3.219835 -1.022513 -2.579098
H 4.562566 0.091488 -2.720048
C -3.839035 -0.321629 2.005160
H -4.562531 0.091450 2.720060
H -3.219805 -1.022553 2.579073

C 4.599448 -1.070364 -0.925149
C 6.014347 -2.493057 1.085777
C 5.997543 -1.104772 -0.906724
C 3.922237 -1.763493 0.088680
C 4.608216 -2.462746 1.074672
C 6.699455 -1.799976 0.076600
H 6.555045 -0.578398 -1.678063
H 2.835285 -1.750016 0.110051
H 4.056038 -2.996595 1.844522
H 7.786632 -1.810501 0.061056
C -4.599443 -1.070374 0.925144
C -6.014375 -2.493028 -1.085787
C -3.922248 -1.763516 -0.088688
C -5.997539 -1.104751 0.906719
C -6.699466 -1.799935 -0.076607
C -4.608243 -2.462750 -1.074682
H -2.835296 -1.750064 -0.110059
H -6.555028 -0.578367 1.678061
H -7.786644 -1.810435 -0.061062
H -4.056078 -2.996609 -1.844533
N 6.705460 -3.242721 2.041554
H 6.205317 -3.393948 2.907042
N -6.705505 -3.242674 -2.041566
H -6.205366 -3.393910 -2.907054
H -7.656763 -2.944564 -2.209876
H 7.656724 -2.944634 2.209865

E = -1224.22195754 Hartree
ZPVE = 277.29532 kcal/mol
Zero-point correction= 0.441898 (Hartree/Particle)
Thermal correction to Energy= 0.467872
Thermal correction to Enthalpy= 0.468816
Thermal correction to Gibbs Free Energy= 0.379087
Sum of electronic and zero-point Energies= -1223.780059
Sum of electronic and thermal Energies= -1223.754086
Sum of electronic and thermal Enthalpies= -1223.753142
Sum of electronic and thermal Free Energies= -1223.842871

Conformer 2 with dispersion
N -4.734220 0.254018 -0.219541
N -4.650195 -0.914704 0.220618
C -3.562282 1.006077 -0.570637
C -1.362696 2.590438 -1.303031
C -2.620607 0.517953 -1.483020
C -3.441307 2.308206 -0.076209
C -2.330725 3.074867 -0.414157
C -1.542819 1.315498 -1.851984
H -2.732923 -0.478541 -1.893762
H -4.204425 2.690607 0.594001
H -2.211567 4.066596 0.013177
H -0.818618 0.934838 -2.566987

C -3.382752 -1.492366 0.570243
C -0.980840 -2.749642 1.302071
C -3.080361 -2.765720 0.078496
C -2.517389 -0.874091 1.479337
C -1.337417 -1.511010 1.847912
C -1.872484 -3.367718 0.416176
H -3.783277 -3.253551 -0.589103
H -2.768013 0.097757 1.887965
H -0.672438 -1.030015 2.559993
H -1.615867 -4.334101 -0.008787
C -0.122403 3.398303 -1.613530
H 0.271138 3.088742 -2.589936
H -0.380250 4.458648 -1.705407
C 0.360575 -3.374716 1.613259
H 0.253661 -4.460391 1.709078
H 0.707472 -3.010037 2.588398
C 0.954629 3.242856 -0.552153
C 2.984959 2.946628 1.406082
C 1.537717 4.355000 0.059331
C 1.400951 1.975340 -0.155941
C 2.397489 1.822295 0.800347
C 2.535423 4.218233 1.022178
H 1.207501 5.352260 -0.220428
H 0.949058 1.090554 -0.594326
H 2.729663 0.826763 1.081474
H 2.973499 5.102086 1.478593
C 1.405660 -3.074396 0.550883
C 3.380027 -2.506196 -1.404673
C 1.658051 -1.760438 0.135484
C 2.153415 -4.095342 -0.040294
C 3.124914 -3.824891 -1.001586
C 2.626003 -1.474094 -0.819676
H 1.075559 -0.947015 0.557654
H 1.975577 -5.126491 0.254579
H 3.693804 -4.639659 -1.441970
H 2.806226 -0.444449 -1.116071
N 4.026698 2.798572 2.323670
H 4.052360 1.905849 2.796920
N 4.393141 -2.217220 -2.320747
H 4.286845 -1.338478 -2.808688
H 4.623892 -2.970991 -2.953261
H 4.138765 3.567913 2.969460

E = -1224.34995225 Hartree
ZPVE = 278.19413 kcal/mol
Zero-point correction= 0.443331 (Hartree/Particle)
Thermal correction to Energy= 0.468948
Thermal correction to Enthalpy= 0.469892
Thermal correction to Gibbs Free Energy= 0.384524
Sum of electronic and zero-point Energies= -1223.906622
Sum of electronic and thermal Energies= -1223.881005

Sum of electronic and thermal Enthalpies= -1223.880060
Sum of electronic and thermal Free Energies= -1223.965428

References

1. Schweighauser, L.; Strauss, M. A.; Bellotto, S.; Wegner, H. A. *Angew. Chem. Int. Ed.* **2015,** *54*, 13436–13439. doi:10.1002/anie.201506126
2. Halgren, T. A. *Encyclopedia of Computational Chemistry*, Vol. 2; Wiley: Chichester, 1998.
3. *Gaussian 16, Revision A.03*: Gaussian, Inc., Wallingford CT, 2016.
4. Becke, A. D. *J. Chem. Phys.* **1993,** *98*, 1372–1377. doi:10.1063/1.464304
5. Lee, C.; Yang, W.; Parr, R. G. *Phys. Rev. B: Condens. Matter Mater. Phys.* **1988,** *37*, 785–789. doi:10.1103/PhysRevB.37.785
6. Krishnan, R.; Binkley, J. S.; Seeger, R.; Pople, J. A. *J. Chem. Phys.* **1980,** *72*, 650–654. doi:10.1063/1.438955
7. Grimme, S.; Antony, J.; Ehrlich, S.; Krieg, H. *J. Chem. Phys.* **2010,** *132*, 154104. doi:10.1063/1.3382344
8. Grimme, S.; Ehrlich, S.; Goerigk, L. *J. Comput. Chem.* **2011,** *32*, 1456–1465. doi:10.1002/jcc.21759
9. Humphrey, W.; Dalke, A.; Schulten, K. *J. Molec. Graphics* **1996,** *14*, 33–38. doi:10.1016/0263-7855(96)00018-5
10. Legault, C. Y. *CYLview;* Université de Sherbrooke, 2009.
